# Synergistic interactions between biogenic organic matter and microbial dynamics during simulated senescent cyanobacterial blooms in freshwater mesocosms

**DOI:** 10.3389/frmbi.2026.1866641

**Published:** 2026-07-13

**Authors:** Noémie Dechaux, Nina Guérin, Najet Thiney, Karine Escoubeyrou, Charlotte Duval, Sarah Fiorini, Charles-Hubert Paulin, Tarik Meziane, Emma Rochelle-Newall, Cécile Bernard, Julie Leloup, Dominique Lamy

**Affiliations:** 1 Sorbonne Université, UMR 7618 CNRS - INRAE - IRD - Univ Paris Cité - UPEC, Institut d’Écologie et des Sciences de l’Environnement de Paris (iEES-Paris), Paris, France; 2 Sorbonne Université, UMR 8067 CNRS, Muséum National d’histoire Naturelle-IRD, Univ Antilles, Laboratoire de biologie des organismes et des écosystèmes aquatiques (BOREA), Paris, France; 3 Sorbonne Université, FR 3724 CNRS, Observatoire Océanologique de Banyuls, Banyuls-sur-Mer, France; 4 Muséum National d’Histoire Naturelle, UMR 7245 CNRS-MNHN, Molécules de Communication et Adaptation des Microorganismes (MCAM), Paris, France; 5 Centre de recherche en écologie expérimentale et prédictive (CEREEP-Ecotron Ile-de- France), Département de Biologie, École Normale Supérieure, CNRS, PSL University, Saint-Pierre-lès-Nemours, France

**Keywords:** cyanobacterial bloom, organic matter, microbial communities composition, free-living, particle-attached, temporal dynamic, freshwater, mesocosms

## Abstract

One of the major consequences of a phytoplanktonic bloom is the massive release of autochthonous organic matter (OM) into the water column, stimulating heterotrophic microbial activity and disrupting the trophic web. To better understand the consequences of such biotic stress, the senescent phase of a bloom was simulated under semi-controlled conditions through the addition of cyanobacterial-derived OM from *Microcystis aeruginosa* and *Aphanizomenon gracile* (30% enrichment as eq C) into lake water mesocosms. By following both the autochthonous OM and the microbial communities during 28 days, we observed that both cyanobacterial-derived OM differed qualitatively (displaying different levels of lability), but enhanced a rapid bacterial mineralization within 2–7 days of incubation. During this early response, we observed an enrichment of Alphaproteobacteria in both the particle-attached (PA) and free-living (FL) fractions, followed by Gammaproteobacteria during the late-response stage. Specifically, in the *Microcystis*-derived OM supply, the emergence and persistence of Bacilli members were detected. At the class level, differences according to cyanobacterial species were also detected, suggesting specific ecological niches within the PA fraction, likely driven by the quality of the cyanobacterial-derived OM pools. Concomitantly, the supply of cyanobacterial-derived organic matter promoted the accumulation of less bioavailable, chemically complex, and persistent compounds, a pattern that was more pronounced for *Aphanizomenon-*derived OM than for *Microcystis*-derived OM. The refractory nature of the remaining OM explained the rapid decline in microbial abundances and activities. The chemical imprint of cyanobacterial-derived OM persisted even after microbial mineralization over the 28-day experiment. Our study highlights that different cyanobacterial OM pools induce specific microbial responses, while also exerting a long-term effect on both OM recycling and microbial community composition.

## 1 Introduction

Cyanobacterial bloom events are widespread globally and are of significant ecological and public health concern because they disrupt the functioning of aquatic ecosystems (Paerl and Huisman, 2008). Enhanced by anthropogenic activities, they are particularly favored in eutrophic conditions in freshwater ecosystems. During a bloom event, the dominance of one or two species first leads to the disappearance of other phytoplankton species, and, by producing harmful toxins, disrupts food webs (Paerl, 1996; Field et al., 1998; Huisman et al., 2018). In parallel, during a bloom event, fresh dissolved organic matter (FDOM) is rapidly and massively released in the water column (Paerl and Otten, 2013; Chen et al., 2025), which stimulates heterotrophic microbial activity (Du et al., 2022; Gao et al., 2023; Chen et al., 2024), and triggers the disappearance of higher trophic levels through oxygen depletion (Paerl and Huisman, 2008). In the context of a bloom event, the pool of phytoplankton-derived organic matter (OM) varies in both quantity and quality according to the identity of primary producers and their physiological states (Biddanda and Benner, 1997; Mangal et al., 2016; Wen et al., 2022). Labile, low-molecular-weight molecules (such as amino acids and fatty acids) are preferentially produced during the growth phase, whereas high-molecular-weight molecules (polysaccharides and proteins) are preferentially produced during the senescence phase (Buchan et al., 2014; Seymour et al., 2017; Camarena-Gómez et al., 2018; Le Moigne et al., 2021).

Heterotrophic prokaryotes (bacteria and archaea) are pivotal actors in the transfer of organic matter through the microbial loop, due to their broad metabolic diversity. In turn, the quantity and the quality of this OM will determine both the fate of enzymatic activities and the structure and the diversity of these microbial communities (Kritzberg et al., 2006; Landa et al., 2014). During the mineralization process, microorganisms will use labile OM first, thereby increasing the proportion of less easily degradable OM, as illustrated for fatty acids by Balzano et al. (2011), and inducing a temporal dynamic of the OM quality. Thus, the fates of the OM and the activities and composition of microbial communities are synergistically linked and can be described as a covariation where both the chemical and microbial activities and diversities promote each other. This synergy is a key process on which the subsequent stages of ecosystem resilience depend (Tanentzap et al., 2019). Furthermore, according to their microbial life strategies as free-living (FL; often defined as the size-fraction <3 µm) or associated with organic particles (PA; defined as the size-fraction >3µm), heterotrophic prokaryotes contribute differentially to the mineralization of dissolved (DOM) and particulate organic matter (POM), thereby partly determining the fate of OM (Grossart, 2010; Martínez-García et al., 2022; Louati et al., 2023; Chen et al., 2025). Moreover, by remineralizing organic particles, PA microbial communities can release dissolved organic matter into the water column, making it available to FL communities. However, this synergy remains poorly explored, especially in the context of cyanobacterial blooms, and its long-term impacts on aquatic ecosystems are not yet well understood.

In this context, the senescent phase of a cyanobacterial bloom is an interesting scenario for exploring whether the biogenically derived OM and heterotrophic microorganisms act synergistically over the long term. During bloom decline and collapse, particulate and dissolved detrital OM is released into the surrounding environment, contributing to water quality deterioration (Nguyen et al., 2005; Louati et al., 2023). This OM enrichment is of major concern because these organic compounds accumulate and enhance heterotrophic respiration. This modifies ecosystem metabolism, leading to net heterotrophy (Staehr and Sand-Jensen, 2007) and potentially to new assemblages of microorganisms. Such quantities of derived OM may also modify the microbial repartition between the free-living and the particle-associated fractions and alter their respective microbial communities (Liu et al., 2024; Xie et al., 2024; Chen et al., 2025). To the best of our best knowledge, the majority of studies investigating the effects of OM release on microbial communities have primarily focused on the bloom growth phase, largely overlooking the senescent stage, and only a few have compared the derived OM from different cyanobacteria genera (Leloup et al., 2013; Liu et al., 2019).

To investigate this phenomenon, we simulated the senescence of two different cyanobacterial blooms and explored the dynamics of both cyanobacterial-derived OM during decay and microbial community dynamics, according to their lifestyle strategy. We selected the common bloom-forming cyanobacteria *Microcystis* and *Aphanizomenon.* The latter is a diazotroph, thus relying differently on nitrogen (N) sources, and can be found under different nutrient-rich conditions (Gobler et al., 2026). The ecology of these cyanobacteria has been extensively studied, demonstrating that they harbor different associated microbiomes (Löptien and Dietze, 2022; Louati et al., 2023; Schweitzer-Natan et al., 2023; Chen et al., 2025). Thus, we explored whether the release of OM derived from these two cyanobacteria would create distinct nutrient-rich environments with different OM composition and lability and differentially alter microbial community structure, potentially promoting the emergence of specialized heterotrophic bacterial taxa. By exploring these interactions, we can gain insights into the microbial processes that occur during post-bloom events and their potential implications for biogeochemical cycles and ecosystem resilience.

## 2 Material and methods

### 2.1 Experimental setup

Approximately 350 L of lake water was collected in January 2018 from the subsurface layer (0.5–1.5m integrated depth) of an artificial lake at the National Experimental Platform for Aquatic Ecology (PLANAQUA) of the Research Center in Experimental and Predictive Ecology (CEREEP), St-Pierre-les-Nemours, France. The water was pre-filtered through a 50-µm Nytex mesh to remove large particles (*e.g.*, leaves and metazoans) and then equally distributed among 12 mesocosms (each 14 L in volume). The mesocosms were stored in a temperature-controlled room in the dark for 4 weeks to inhibit photosynthesis by phytoplankton and allow degradation of the majority of the labile organic matter present in the water at the time of sampling. During these 4 weeks, the temperature was gradually increased from 13 °C to 23 °C.

The non-axenic strains of *Aphanizomenon gracile* (PMC 644.10) and *Microcystis aeruginosa* (PMC 810.12) were obtained from the cyanobacteria and microalgae collection at MNHN-Paris, France (https://www.mnhn.fr/en/cyanobacteria-and-eukaryotic-microalgae-collection). They were cultivated in standard Z8 medium (Hamlaoui et al., 2022), without agitation, at 25 °C with an 18:8 h (day/night) photoperiod and a light intensity of 10 μmol photons m^-2^ s^-1^. First, the cellular abundance was measured by optical density (600 nm) to collect cultures during the exponential phase. Each culture was then inactivated by microwave radiation (800W, 3*3 min; Keller et al., 1988) to mimic senescent cyanobacteria-derived OM sources. As the cyanobacterial cultures were non-axenic, the OM collected for the experiment should be considered cyanobacteria-derived OM, together with the mineral nutrients present in the cultures. Then, particulate and dissolved carbon (C) and N contents were determined in the inactivated non-axenic cultures and in the freshwater lake water (**Supplementary Table 1**). The volume added under each cyanobacterial treatment was calculated in order to achieve enrichment to 30% of the existing C pool in the freshwater lake water. To obtain 161 µM eq C cyanobacteria-derived OM (dissolved and particulate organic carbon) in each treatment, a fixed volume of each cyanobacteria-derived OM source (1,100 mL for *A. gracile* and 665 mL plus 435 mL of sterile distilled water for *M. aeruginosa*) was then added to four replicate mesocosms (n = 4 per treatment: A and M). The control treatment (C) consisted of four additional mesocosms maintained under the same conditions without OM supply but amended with 1,100 mL of sterile distilled water.

### 2.2 Sampling strategy

Temporal sampling was performed just before OM addition (d0) and after 2 (d2), 7 (d7), 14 (d14), and 28 (d28) days of incubation. Mesocosms were covered to prevent evaporation while allowing gas exchanges with the atmosphere. Aquarium circulation pumps ensured mixing of the water column throughout the experiment in all mesocosms. Daily immersion of a multiparameter probe (YSI Exo2) was performed to measure temperature, pH, O_2_ saturation, and Chlorophyll *a* (Chl-*a*) values. At each sampling date (n = 5), 2 L were sampled in acid-washed (10% HCl) and MilliQ-water-rinsed carboys. At the of the experiment, 1/3 of the initial volume (10 L) remained in each mesocosm. Temperature varied from 20.6 ± 0.3 °C to 2.4 ± 0.7 °C throughout the experiment.

#### 2.2.1 Sampling for the chemical compartment

From the raw water samples, technical duplicate samples (30 mL each) were immediately frozen (–20 °C) for total hydrolyzable amino acids (THAA) analysis. Technical duplicate samples (300 mL each) were filtered through pre-combusted (450 °C overnight) 0.7µm porosity glass fiber filters (GFF - Whatman). The filters, containing the particulate fraction of the OM, were kept for fatty acid analyses. The eluates, containing the dissolved pool, were collected (30 mL in duplicate for each parameter) for: (i) nutrient analyses in polyethylene tubes (NH_4_^+^, NO_3_^--^NO_2_^-^, and PO_4_^2-^ concentrations); (ii) dissolved organic carbon (DOC) concentration in glass tubes after preservation with 36 μL phosphoric acid (H_3_PO_4_ 85%); and (iii) colored dissolved organic matter (cDOM) analyses in amber bottles sealed with Teflon-lined caps. The filters and the eluates were stored at –20 °C.

#### 2.2.2 Sampling for the microbial compartment

From the raw water samples, technical duplicate samples (1 mL each) for flow cytometry analysis were fixed with glutaraldehyde (0.5% final concentration) and kept in the dark at 4 °C for 30 min before being frozen at –80 °C. Technical triplicate samples (100 mL) were filtered through a 3 µm porosity filter (Nuclepore Polycarbonate filter; Whatman) to collect the particle-attached fraction (PA), followed by filtration through a 0.2 µm porosity filter (Nuclepore Polycarbonate filter; Whatman) in order to concentrate the free-living fraction (FL). Filters were immediately frozen in liquid nitrogen and stored at –80 °C until DNA extraction.

### 2.3 Nutrient analyses

Concentrations of dissolved inorganic nitrogen (NH_4_^+^, NO_3_^--^NO_2_^-^, DIN), phosphorus (PO_4_^2-^, DIP), dissolved organic nitrogen (DON), and phosphorus (DOP) were determined in duplicate using an automated colorimetric procedure as described by Holmes et al. (1999) at the MIO-PACEM analytical platform (https://www.mio.osupytheas.fr/fr/plateformes-de-recherche/ptf-analytique-de-chimie-des-environnements-marins-pacem). Total dissolved nitrogen (TDN) was calculated by adding DIN and DON concentrations, while total dissolved phosphorus (TDP) was calculated by adding DIP and DOP concentrations.

Dissolved organic carbon (DOC) concentration was measured on a Shimadzu TOC V_CPH_ analyzer as described by Rochelle-Newall et al. (2014), and particulate organic carbon and nitrogen (POC and PON, respectively) concentrations were determined by gas chromatography after combustion using an elemental CHN analyzer, as described by Sharp (1974), at the iEES-Paris analytical platform (https://iees-paris.fr/technical-platforms/ecogeochemistry/). At d0, right before the addition of cyanobacterial-derived OM, mean DOC concentrations in the mesocosms amounted to 514 ± 36 µM, while mean POC concentrations did not exceed 51± 12 µM.

### 2.4 Dissolved organic matter composition and quality

#### 2.4.1 Optical properties of colored dissolved organic matter

Spectrophotometric and fluorometric methods were used to approximate DOM composition (Rochelle-Newall and Fisher, 2002b; Rochelle-Newall et al., 2014; Li and Hur, 2017). For colored dissolved organic matter (cDOM) measurements, 30 to 50 mL filtered samples were stored in pre-cleaned 125-mL amber glass bottles sealed with Teflon-lined caps. After collection, the samples were stored frozen (–20 °C) until measurement. Before the optical measurements, the samples were thawed slowly to room temperature and re-filtered through 0.2-μm syringe filters (Sartorius Minisart NML). The cDOM absorption was measured using a spectrophotometer (Analytica Jena Specord 205 UV–VIS) from 200 to 750 nm with a 10-cm quartz cell, using Milli-Q water as the blank, and was expressed in m^-1^. The A_220_/A_254_ ratio and the A_210_/A_254_ ratio were calculated because they have been associated with DOM polarity (Erlandsson et al., 2012) and DOM aromaticity (Huang et al., 2016), respectively. The carbon normalized absorption at 254 nm (SUVA_254_) was computed by dividing the UV absorbance at 254 nm by the concentration of DOC (mg C.L^-1^; Hood et al., 2006) and was used as a proxy for the aromatic content of DOM (Weishaar et al., 2003). The spectral slope ratio *Sr* was calculated as the ratio of the slope of the shorter UV wavelength region (275–295 nm) to that of the longer UV wavelength region (350–400 nm) (Helms et al., 2008), and was obtained using linear regression on the log-transformed spectral ranges (Yamashita et al., 2010). We reported the ratio 1/*Sr* as a proxy for the apparent molecular weight (MW) of cDOM (Helms et al., 2008), where higher values of 1/*Sr* suggest higher apparent MW. Finally, fluorescence measurements were performed using a Turner Trilogy^®^ fluorometer equipped with a UV module insert (excitation = 350 nm and emission wavelength = 410–450 nm) according to Rochelle-Newall et al. (2014), and values were expressed in Turner fluorescence units (TFlU). The carbon-normalized cDOM fluorescence (Fluo/DOC, in TFlU per mg C.L^-1^) was used as a proxy for the biodegradability of DOM, with higher values generally associated with lower bioavailability for microbial processes (Blough and Del Vecchio, 2002; Rochelle-Newall and Fisher, 2002a; Rochelle-Newall et al., 2018).

#### 2.4.2 Fatty acid analysis

Fatty acids (FA) were analyzed on the particulate fraction (>0.7 µm) following a slightly modified Bligh and Dyer (1959) protocol, as described by Meziane et al. (2007). Fatty acid methyl esters (FAMEs) in chloroform were quantified by gas chromatography (GC; Agilent 8890) coupled with a flame ionizer and then identified by comparing chromatograms with those generated by a GC coupled to a mass spectrometer (Agilent 5977B), a commercial standard (Supelco^®^ 37 component FAME mix), and the laboratory reference library. Fatty acid concentrations were calculated using the added 23:0 (internal standard) known concentration, assuming that they all undergo equivalent losses. We reported the values as % of total FA, and calculated the sum of saturated fatty acids (SFA), the sum of branched fatty acids (BFA) as bacterial markers, and the ratio PUFA/MUFA (proportion of polyunsaturated FA/monounsaturated FA), where higher values suggest relatively fresher and more labile autochthonous organic matter (Canuel, 2001).

#### 2.4.3 Amino acid analysis

Total hydrolyzable amino acids (THAAs) were analyzed by high-performance liquid chromatography (HPLC) after liquid-phase acid hydrolysis according to Escoubeyrou and Tremblay (2014). Specifically, a high volume (100 µL) of undiluted samples was injected on a robust hybrid C18 column. HPLC analyses were performed using a Thermo Scientific Ultimate 3000 HPLC system equipped with an autosampler and a fluorescence detector (excitation at 335 nm and emission at 450 nm). The separation was achieved using a Phenomenex Gemini C18 column (250 x 4.6 mm, 5 µm, 110 Å) and a Security Guard column (4 x 3 mm).

Among the 18 amino acids (AAs) targeted, only 16 could be quantified (above the detection limit): glutamic acid, glycine, leucine, alanine, aspartic acid, γ-aminobutyric acid, phenylalanine, lysine, arginine, threonine, serine, valine, histidine, ornithine, β-alanine, and isoleucine. Degradation index (DI) was calculated using AA composition according to Dauwe et al. (1999) and adapted to aquatic DOM (Davis et al., 2009; Kaiser and Benner, 2012), as an indicator for the DOM degradation state. This index links the AA composition to OM diagenesis and is used to estimate DOM quality based solely on chemical composition. The index considers the whole suite of AA in the calculation and allows different samples to be directly and quantitatively compared. Negative DI values are indicative of degraded OM, whereas positive DI values are indicative of fresh OM. In addition, the quantity (mol %) of the non-protein AAγ-aminobutyric acid and β-alanine, together with the ratio (in %) of glycine (mol %)/(glycine + serine + threonine, mol%), were used as indicators of OM degradation processes (Peter et al., 2012).

### 2.5 Flow cytometry analysis

Cellular abundances of virus-like particles, heterotrophic prokaryotes, and micro-eukaryotes were determined by the CYSTEM platform (https://partner.uca.fr/poles-de-competences/microscopie-imagerie/cytometry-sort-transmission-electronic-microscopy-cystem) using a FACS Calibur flow cytometer (Becton Dickinson, Franklin Lake, NJ, USA) equipped with an air-cooled laser providing 15 mW at 488 nm. Briefly, extracted samples were diluted with 0.2 μm prefiltered TE buffer (10 mM Tris-HCL and 1 mM EDTA, pH 8) and stained with SYBR Green I (10,000-fold dilution of commercial stock; Molecular Probes, Eugene, OR, USA). The mixture was incubated for 5 min, heated for 10 min at 80 °C in the dark, and cooled for 5 min prior to analysis. Prokaryotic and virus-like cells differing in fluorescence intensity were detected by their signature in side scatter versus green fluorescence (530 nm wavelength, fluorescence channel 1 of the instrument plot). Flow cytometry list modes were analyzed using CellQuest Pro software (BD Biosciences, version 4.0). A blank was routinely examined to control for contamination of the equipment and reagents.

### 2.6 Microbial metabolic profiling

The metabolic profile of microbial communities was assessed using the Biolog^®^ EcoPlate™ method (Garland et al., 2001). At sampling dates d0, d14, and d28, raw water (150 µL) was inoculated into each well of the microplates (Alliance Bio-expertise, Guipry-Messac, France) and then incubated in the dark at 23 °C. Absorbance was measured at 595 nm every 24 h for 9 days using a microplate reader (Bio-Rad Laboratories, Model 680). After data trimming (absorbance-threshold value of 0.25), the average well-color development (AWCD) was determined as: AWCD = [Σ (Abs_substrate_ - Abs_control_)]/Σ (Abs_all subtrates_); where Abs_substrate_ represents the absorbance value of a given well, Abs_control_ is the absorbance of control wells (containing no substrate), and Abs_all subtrates_ is the sum of the absorbance of all substrates. The functional richness (FR) was expressed as the number of different substrates used by the microbial community. The nitrogen use index (NUSE) was expressed as the relative absorbance of nitrogen-containing substrates (Sala et al., 2006). All data used were acquired at the seventh time point to ensure that all enzymatic reactions were detected, allowing for comparisons between treatments.

The exoproteolytic activity (EPA) was measured using the fluorogenic substrate analog L-leucine-methylcoumarinylamide (Leu-MCA; Hoppe, 1993). The substrate was added to 3 mL subsamples and incubated for 1.5 h at *in situ* temperature and low natural light intensity. At the end of the incubation period, the exoenzymatic cleavage activity was stopped using sodium dodecyl sulfate (1% final concentration). Controls in duplicates were processed similarly, except that the stopper solution was added before the substrate. Cleavage of Leu-MCA results from the exoenzymatic activity and is linearly related to the MCA fluorescence. Saturation curves were generated to determine the saturating substrate concentration, and a final concentration of 350 µM was used for all samples. Since the substrate was saturating in our study, the results corresponded to the maximum potential α-aminopeptidase exoenzymatic activity. The fluorescence was measured using a Varian Cary Eclipse fluorescence spectrophotometer (excitation/emission of 380/440 nm) and transformed to cleavage activity using a standard curve established with different concentrations of the MCA fluorochrome.

### 2.7 Microbial composition

Total DNA was extracted from a total of 128 filters (4 filters per date, treatment, and size fraction, in addition to 4 replicates of each cyanobacterial culture) using the AllPrep DNA/RNA kit (Qiagen), according to Hugoni et al. (2013). Isolated DNA was quantified using fluorimetry (NanoDrop, ND-800, Thermo Scientific, USA).

Amplification of the V3–V4 region of the 16S rRNA was performed using the universal primer 343-F (5’- TACGGGAGGCAGCAG -3’) and the bacteria/archaea specific primer 790-R (5’- CCA GGGTATCTAATCC -3’). Primers were modified by adding Illumina adapter barcodes to both ends. Each PCR was performed in a final volume of 50 μL, containing 5 μL of 10X buffer, 2 μL of bovine serum albumin (BSA 2mg.mL^-1^), 1 μL of Taq Platinum™ (Invitrogen, 5 U.μL^-1^), 1 μL of dNTP (10 mM of each), and 5μL of each primer (1 μM). The amplification conditions consisted of initial denaturation at 95°C for 3 min, followed by 25 cycles at 95°C for 30 s, 55°C for 30 s, and at 72°C for 30 s, and a final elongation of 5 min at 72°C. The PCR products were run on a 1% agarose gel electrophoresis. The amplicons were subsequently processed by Eurofins (Konstanz, Germany) for the library preparation (addition of barcode, purification, and equimolar mix) for Illumina Sequencing Technology (paired-end read length: 2 × 300 bp).

### 2.8 Bioinformatic analyses

A total of 125 samples were successfully sequenced, and reads presented a Phred quality score above a threshold of 30 (FastQC software; https://www.bioinformatics.babraham.ac.uk/projects/fastqc); three samples failed (PA-d0-A-R2, PA-d0-M-R1, and PA-d2-C-R4, see **Supplementary Table 2**).

Data were processed through the FROGS pipeline (Find Rapidly OTU with Galaxy Solution, release 4.1.0), implemented on a galaxy instance (release 21.05) (https://galaxy.migale.inra.fr/; (Escudié et al., 2018). Following preprocessing (merging overlap, size, and without N), a total of 9,804,461 assembled reads were analyzed. Reads were clustered using the SWARM algorithm (2.1.5), which applies a single linkage clustering on sequences. The fastidious option was used, with clustering of nearly identical amplicons iteratively with an aggregation distance of 1, as recently recommended (FROGS 3.2 version release 2021). Chimeras were then removed using VSEARCH (Rognes et al., 2016). Low-abundance ASVs (<5 reads) were removed from the dataset (filtering step). At the sample level, datasets were rarefied to the lowest number of reads (15,613 reads). Taxonomy affiliation of each ASV was performed using the BLASTn+ tool (version 2.10; Camacho et al., 2009) against the SILVA 138-16S pintail 100 database (Pruesse et al., 2007), and only the best BLAST hits with the same score are reported.

All the nucleotide reads have been deposited in the SRA database (Sequence Reads Archive) under accession number PRJNA1456822 (http://www.ncbi.nlm.nih.gov/bioproject).

### 2.9 Statistical analyses

All statistical analyses were performed using R (versions 4.2.3 and 3.3.6; R Development Core Team, http://www.R-project.org). The Phyloseq (1.36.0; McMurdie and Holmes, 2013) and Microbiome (1.14.0; Lahti and Shetty, 2012–2019) packages were used to describe microbial community composition and diversity. Plots were generated using the packages ggplot2 (3.4.2; Wickham, 2016) and Phyloseq. In order to identify significant temporal groupings (p-value < 0.05 and 999 permutations) within both free-living and particle-associated microbial communities, a SIMPROF analysis (similarity profiling with no *a priori*, Clustsig package 1.1; Clarke et al., 2008) was performed, based on a Bray–Curtis distance matrix.

A metrical dimensional scaling (MDS), based on the Bray–Curtis distance matrix, was used for the ordination of the microbial communities. Differences according to the treatments (with or without cyanobacterial-derived OM supply, in addition to OM origin) and the time response were statistically tested using permutational PERMANOVA (9,999 permutations), with ADONIS (vegan 2.6.2; Oksanen et al., 2022; see **Supplementary Table 3A**). Pairwise comparisons were then performed using a Bonferroni adjustment in order to test whether microbial communities differed significantly according to cyanobacterial-derived OM addition within each fraction (FL and PA) and across the different time periods (initial, early, and late) (**Supplementary Tables 3B, C**).

Diversity indices (species richness, Shannon, Simpson, and Evenness) were calculated using Phyloseq. Differences were tested within each fraction according to cyanobacteria-derived OM supply across the different time responses using a linear mixed model (LMM). The formula was as follows: Y ~ treatment * date + (1 | mesocosm) or Y ~ treatment * date + (date | microcosm) using the *lmer* function (from lmerTest v3.1-3; Kuznetsova et al., 2017) and *lsmeans* function (from Emmeans v1.10.2; Lenth, 2016). Different models, including or excluding fixed effects and incorporating random effects on the slope and/or intercept, were compared using the *anova* function (Chambers and Hastie, 1992) to assess their relative fit. Model selection was based on the Akaike Information Criterion (AIC) and the Bayesian Information Criterion (BIC), with the model exhibiting the lowest values for both criteria being retained. Pairwise comparisons were adjusted using the Holm adjustment method (**Supplementary Table 4**).

The relative abundances of the major taxonomical classes within each fraction (**Supplementary Tables 5A, B**) were compared using analysis of variance (ANOVA), followed by Tukey’s HSD *post-hoc* tests when necessary. These analyses evaluated differences associated with cyanobacterial-derived OM supply across the different time responses (**Supplementary Tables 5C, E**) and differences between cyanobacterial species when considering only OM-amended samples (**Supplementary Tables 5D, F**). The ASVs contributing most strongly to the dissimilarity among communities were identified using the SIMPER function (vegan 2.6.2; Oksanen et al., 2022) with the criterion of up to 70% of the cumulative explained dissimilarity (adjusted p-value < 0.1). Then, the ASVs were screened for significant differential expression using DESeq2 (p_adj_ < 0.01; 1.42.0; Love et al., 2014; **Supplementary Table 6**).

Differences in inorganic nutrient concentrations, OM concentrations, and OM quality were tested according to cyanobacteria-derived OM supply across the different time responses using a linear mixed model analysis (LMM, **Supplementary Table 7**), as previously described.

Multiple factor analysis (MFA; Escofier and Pagès, 1994) was conducted using the *ade4* package to identify common patterns among different groups of variables characterizing the chemical properties of inorganic and organic materials of water at the initial state and following cyanobacteria-derived OM addition. These groups of variables included dissolved organic and inorganic nutrient concentrations, fatty acid and amino acid profiles, and optical properties of cDOM, all measured on the same set of samples. Briefly, the overall analysis consisted in performing separate principal component analyses (PCA) on each group of variables before combining them into a single dataset. The contribution (in %) of each variable to the first and second dimensions was calculated as the ratio of cos2 of each variable divided by the sum of cos2 values for all the variables, multiplied by 100. A variable was considered to contribute substantially to a dimension when its contribution exceeded the average expected contribution of the 17 variables (1/17) x 100 = 5.88%.

Relationships between bacterial community structure and environmental variables were explored by a redundancy analysis (vegan 2.7.3). Data were first standardized using a Hellinger transformation (decostand function). The adequate model was selected using the OrdiR2Step function (1,000 permutations; vegan 2.7.3) to identify the appropriate environmental variables to be included (**Supplementary Table 8** and **Supplementary Figure 10**). The redundancy analysis was performed using the following formula: Y~NH4 + TDP + FLUO/DOC + DOC + Aromaticity + SUVA254 + TDN + Prok_abundance, on both the whole dataset and a reduced dataset (set of ASVs with a relative abundance > 0.1% of the whole dataset). Both analyses produced similar results.

## 3 Results

### 3.1 How does cyanobacterial-derived organic matter modify the microbial compartment?

**Figure 1:**
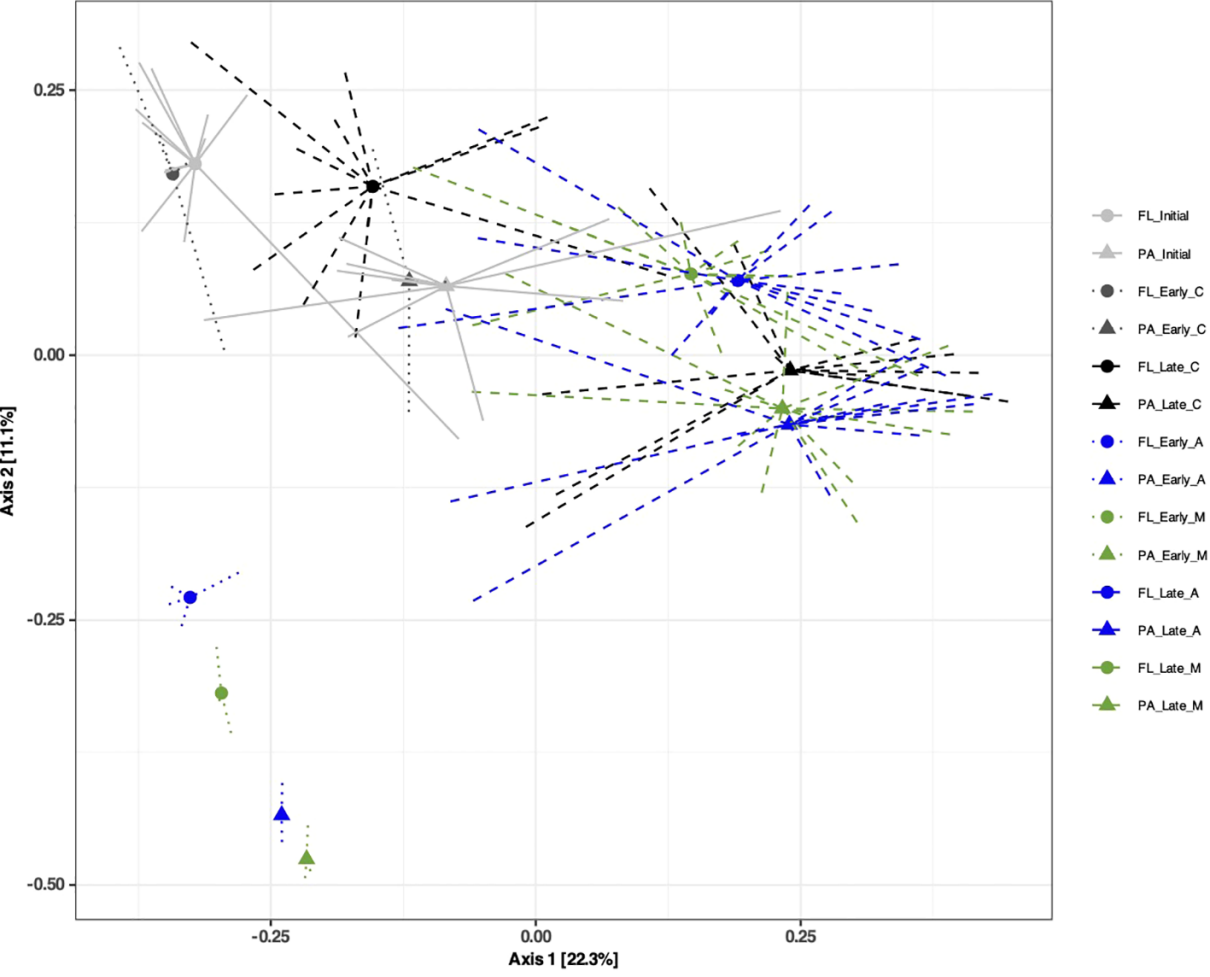
Temporal dynamics of the microbial communities. MDS analysis was performed on both bacterial communities from the free-living (FL, dot) and particle-associated (PA, triangle) fractions for the control condition (gray, C), *Aphanizomenon*-derived OM (blue, A), and *Microcystis*-derived OM (green, M), according to the initial (solid line), early response (dotted line), and late response (long-dashed line) periods. In the spider plot, the forms correspond to the position of the centroid of each condition, and the ends of the lines correspond to the position of each sample.

The structures of the microbial communities showed a clear temporal response in both the FL and PA fractions (**Figure 1**, p-value < 0.0001, **Supplementary Table 3**), as further supported by the similarity profile analysis, which revealed a strong pattern after two days of OM supply (shown as d2; **Supplementary Figure 1**, p-value < 0.05). This highlighted two time responses of the microbial compartment, which were thus defined with data from d2 as “early-response” and data from d7, d14, and d28 as “late-response” (**Figure 1**; p-value < 0.0001; **Supplementary Table 3**).

**Figure 2:**
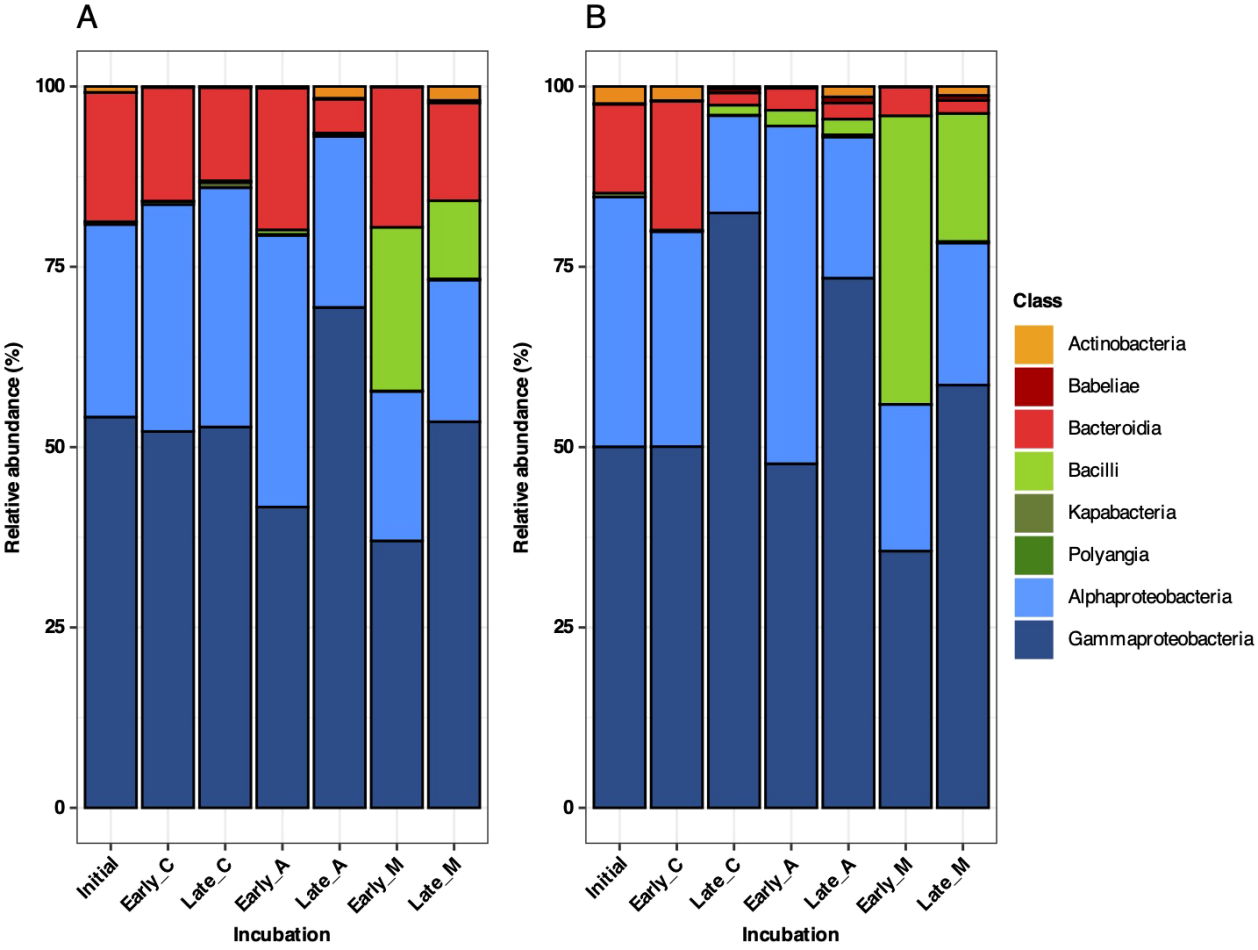
Temporal dynamics of the bacterial taxonomic composition from the free-living **(A)** and particle-associated **(B)** fractions. Data are presented as relative abundances (%) of the dominant ASVs (> 0.1% of the whole dataset) at the class level, according to the time-response period (initial, early response, and late response) and the derived-OM supply: *Aphanizomenon*-derived OM (A), *Microcystis*-derived OM (M), and Control (C).

Under the control conditions, without cyanobacterial-derived OM supply, the structure of the initial communities did not differ statistically from that of the early-response communities in either the FL or PA fraction, but differed from the late-response communities (**Figure 1**; **Supplementary Tables 3B, C**, p-value < 0.010). A similar trend was observed for the diversity indeces (Shannon, Simpson, and Evenness), which decreased during the late-response condition in the PA communities (**Supplementary Figure 2**, **Supplementary Table 4**), while prokaryote and micro-eukaryote abundances (measured by flow cytometry) remained stable (**Supplementary Figure 3**). Regarding taxonomic composition, statistical differences were only detected for the PA fraction among the initial, early, and late conditions (**Figures 2A, B**; **Supplementary Table 5**). A strong, significant increase of Gammaproteobacteria (from 50.1% to 82.5%, p-value < 0.0001 and 0.0064) was observed, concomitant with the decrease in Bacteroidia (17.9% to 1.72%, p-value < 0.1) and Alphaproteobacteria (34.7% to 13.5%, p-value < 0.05) during the late response condition. Altogether, these data illustrate how the microbial communities changed in the absence of OM supply in the mesocosms (control conditions).

In contrast, when cyanobacterial-derived OM was supplied (regardless of its origin), strong differences between the control conditions and both the early response and late response communities were detected, for both the PA and FL fractions (**Figure 1**, p-value < 0.01, **Supplementary Table 3**), highlighting the quick impact of the OM supply. The distinction between the control condition and the late-response condition was less pronounced, implying a return to the initial conditions after 7 days. The diversity indices showed contrasting results between the two fractions. While significant differences persisted between initial and late response in the FL fraction for both cyanobacterial-derived OM treatments (p < 0.05, **Supplementary Table 4**), no significant difference was observed for any of the indices in the PA fraction (p > 0.05, **Supplementary Table 4**).

Concomitantly, cyanobacterial-derived OM supply induced an increase in prokaryote and micro-eukaryote abundance during the early response at d2 (**Supplementary Figure 3**). Additionally, an increase in virus-like particles (VLP) abundance was detected at d7 of (p-value < 0.01), coinciding with decreases in prokaryote and micro-eukaryote abundances. This led to higher ratios of virus-like particles to prokaryotes (VLP: Prok) and virus-like particles to micro-eukaryotes under late-response conditions (26.6 ± 3; **Supplementary Figure 3**), suggesting that the OM supply modified the balance of the abundances of VLP and prokaryotes. No statistical differences in VLP, prokaryote, and micro-eukaryote abundances were detected according to the two cyanobacteria species.

Similarly, when the cyanobacterial-derived OM was added, the community composition during both the early- and late-response conditions showed statistical differences, although the same taxonomical classes remained dominant (**Figure 2**; **Supplementary Table 5**). In both the FL and PA fractions, the early response was characterized by a decrease in Gammaproteobacteria, followed by an increase during the late-response condition (p-value < 0.05, **Supplementary Table 5E**). In the PA fraction, the decline of Bacteroidia was also observed during the early-response (p-value < 0.005), and persisted during the late response (**Supplementary Table 5E**). A similar dynamic for both Gammaproteobacteria and Bacteroidia was observed in the FL fraction during the late-response condition (p-value < 0.05, **Supplementary Table 5C**). The cyanobacterial-derived OM supply also led to the emergence of Bacilli in both the PA and FL fractions, and these persisted in the late-response communities (**Figure 2**; **Supplementary Tables 5C, E**).

Although the origin of the cyanobacterial-derived OM supply, *Microcystis* or *Aphanizomenon*, did not statistically impact the microbial structure, as shown in **Figure 1** (see **Supplementary Table 3**), we detected specific effects at the class level, both for the FL and PA fractions (**Figure 2**). The *Aphanizomenon*-derived OM supply led to an increase in Alphaproteobacteria abundance at d2 in the PA fraction (p-value < 0.01, **Supplementary Table 5F**). The *Microcystis*-derived OM supply led to a dominance and persistence of Bacilli in both the PA and FL fractions, with relative abundance ranging from 10.86% to 40.1% (**Supplementary Tables 5A, B**, p-value < 0.01), while it represented only 0.1% to 1.38% of the communities under control conditions, and 2.17% to 2.15% when *Aphanizomenon*-derived OM was supplied. This class was not detected within the cyanobacterial-derived OM prior to its addition (**Supplementary Figure 4**).

**Figure 3:**
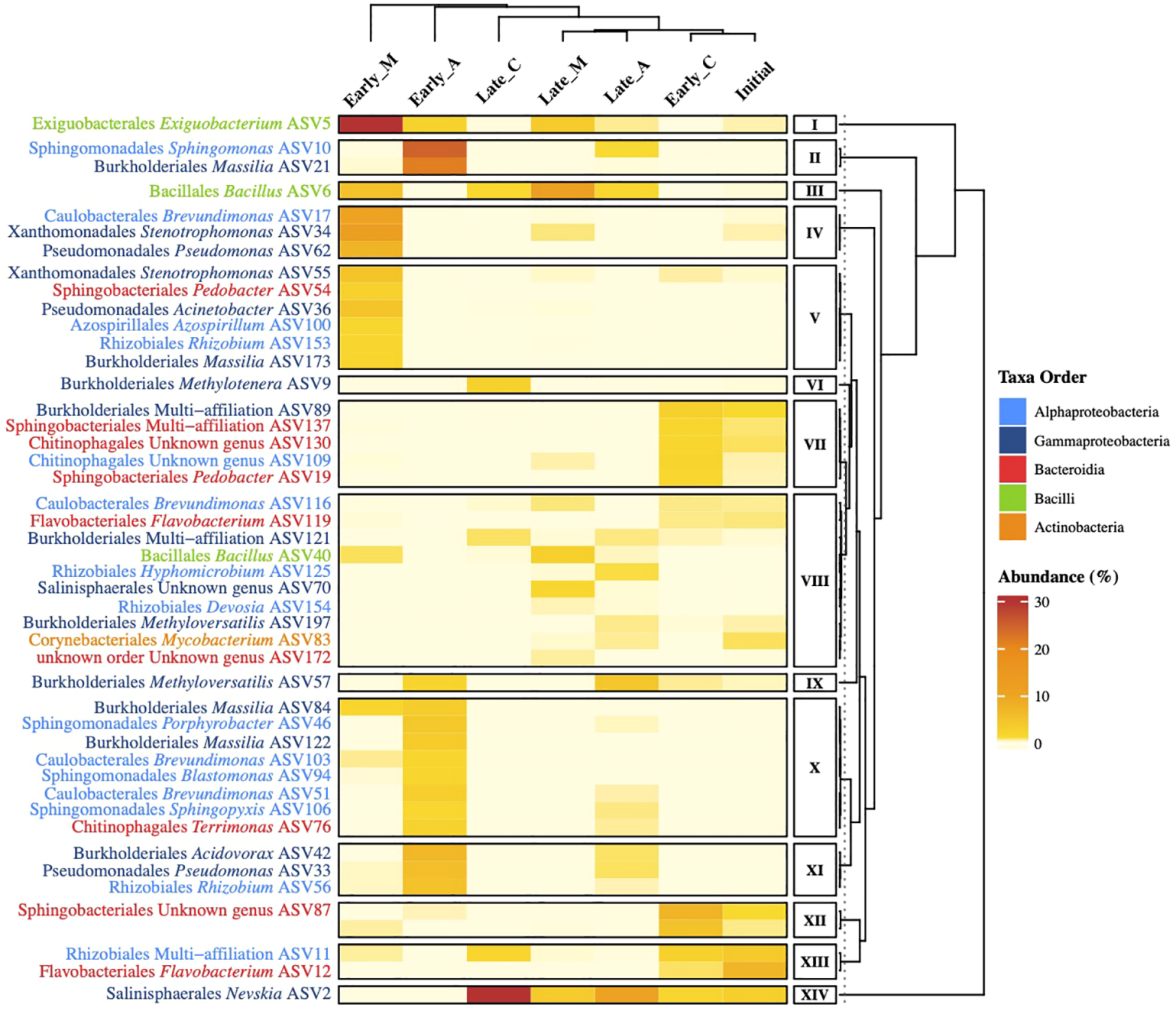
Heatmap of the selected ASVs explaining the dissimilarity between the particle-associated communities. Data are presented at the genus taxonomic level and expressed as percentages of the total read abundance. Only ASVs with a significant differential expression between conditions are represented (p-value significance < 0.01).

Finally, we investigated the contribution of specific ASVs responsible for the strong pattern observed in the PA fraction using SIMPER and differential expression analyses (p-value < 0.01), which identified 45 ASVs (**Supplementary Table 6**). The temporal dynamics of these 45 ASVs (**Figure 3**) showed a clear distinction of taxa group, with an overall dominance by members of the Alphaproteobacteria and Gammaproteobacteria. Under control conditions, the majority of the ASVs showed a stable pattern over time, although several Bacteroidia-associated ASVs (including members of the Sphingobacteriales, Rhodobacterales, Chitinophagales, and Flavobacteriales) remained relatively abundant and were almost undetected in all the other conditions. The early response in the *Aphanizomenon-*derived OM condition was characterized by a strong increase in ASVs affiliated with *Massilia* (Burkholderiales) and *Sphingomonas* (Sphingomonadales), corresponding to cluster II, and to a lesser extent ASVs from clusters X and XI (**Figure 3**), which included members belonging to Burkholderiales, Sphingomonadales, Rhizobiales, and Pseudomonadales. In the *Microcystis-*derived OM conditions, we detected a strong increase of ASVs of *Bacillus* and *Exiguobacterium* (Bacilli; cluster I), followed by *Stenotrophomonas, Brevundimonas*, and *Pseudomonas* ASVs (Xanthomonadales, Caulobacterales, and Pseudomonadales, respectively), in clusters III, IV, and V, respectively (**Figure 3**). Collectively, these results highlight that differences were detected with different ASVs at the same taxa, family, and class level. Moreover, these taxa were still present in high abundances during the late-response conditions. As highlighted in **Figure 2**, the differentiation with the *Microcystis-*derived OM supply is supported by the dominance of Bacilli (*Bacillus*) and Actinobacteria (*Mycobacterium*), which remained stable over time during the early- and late-response conditions.

### 3.2 Does the senescence of cyanobacteria fingerprint the chemical compartment?

**Figure 4:**
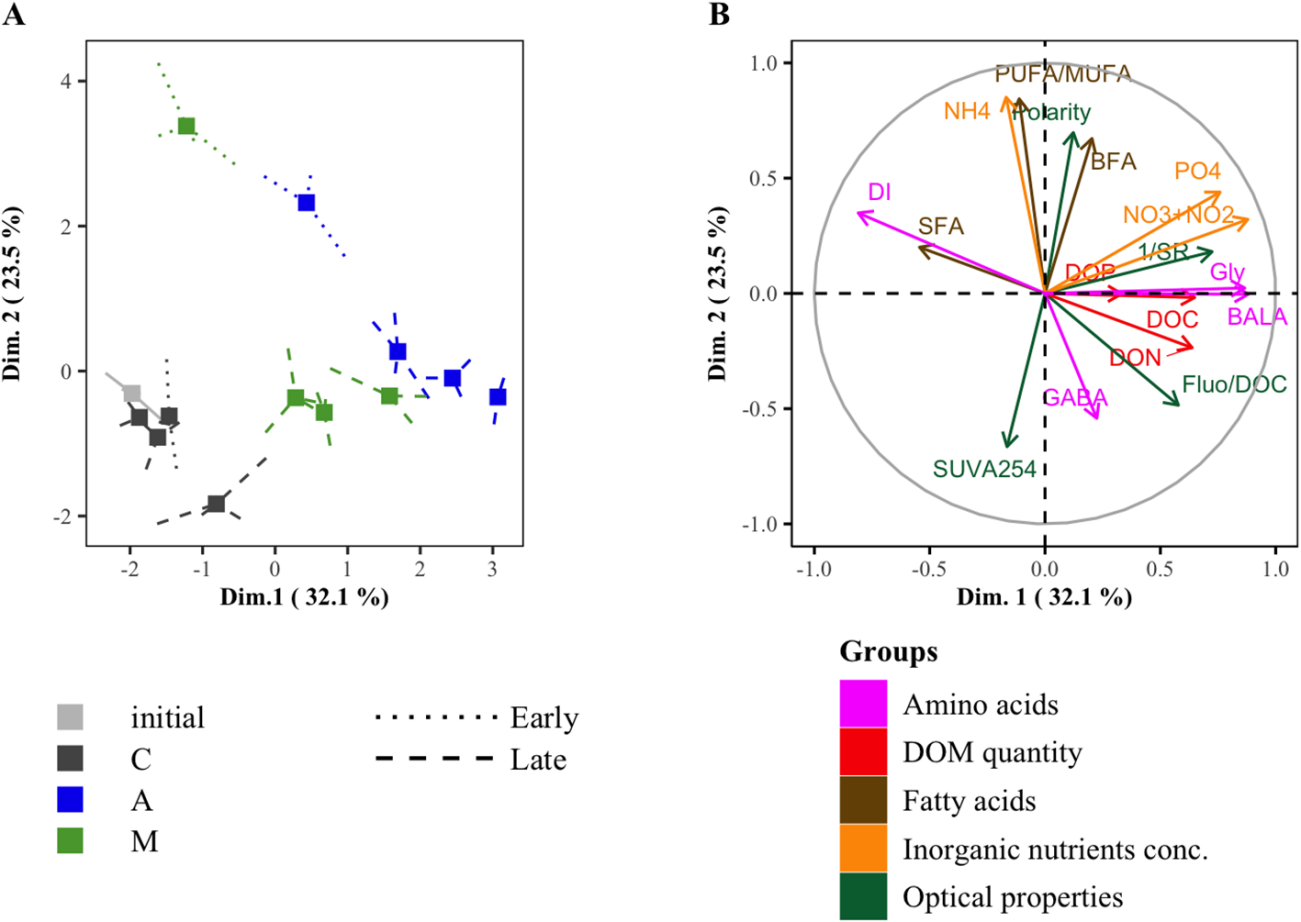
Multiple Factor Analysis of the chemical properties of inorganic and organic nutrients. Individual sample map **(A)**, according to the time-response period (initial, early response, and late response) and the derived-OM supply: *Aphanizomenon*-derived OM (A), *Microcystis*-derived OM (M), and Control (C). In the spider plot, the forms correspond to the position of the centroid of each condition, and the ends of the lines correspond to the position of each sample. Correlation circle **(B)** of the variables, colored according to the following categories: amino acids, fatty acids, inorganic nutrients, DOM quality, and optical properties.

As observed for the microbial communities, the chemical compartment also exhibited early and late responses, both in the concentrations of organic and inorganic nutrients (**Supplementary Figure 6** and **Supplementary Table 7**) and in OM chemical composition and quality, as illustrated by the spatial discrimination shown in **Figure 4**.

First, the cyanobacterial-derived OM supply induced a peak in inorganic nutrient concentrations (**Supplementary Figures 6A, B**), resulting in higher nitrate plus nitrite (NO_3_^-^ + NO_2_^-^) and phosphate (PO_4_^2-^) concentrations than in the control condition (p < 0.0001, **Supplementary Table 7A**). These concentrations then remained stable over time and were higher in *Aphanizomenon* than in *Microcystis* samples (p-value< 0.001, **Supplementary Table 7A**). Additionally, a peak in NH_4_^+^ concentration was detected during the early response (p < 0.0001 for C *vs* A and C *vs* M, **Supplementary Table 7A**), before rapidly returning to the control level during the late response (p > 0.05, **Supplementary Table 7A**). Peaks in DON and DOP concentrations were detected after a time lag, at d7, for A and M conditions, respectively (p < 0.05, **Supplementary Table 7B**). Few significant differences were observed for DOC over time, mostly between d2 and d28 for *Aphanizomenon-*derived OM (**Supplementary Figures 6D, E** and **Supplementary Table 7B**), suggesting rapid mineralization. This is also supported by a peak of exo-proteolytic activity at d2 (p < 0.0001 for both A and M compared to the control), followed by a rapid decrease to the levels observed under the initial and control conditions (**Supplementary Figure 7**).

**Figure 5:**
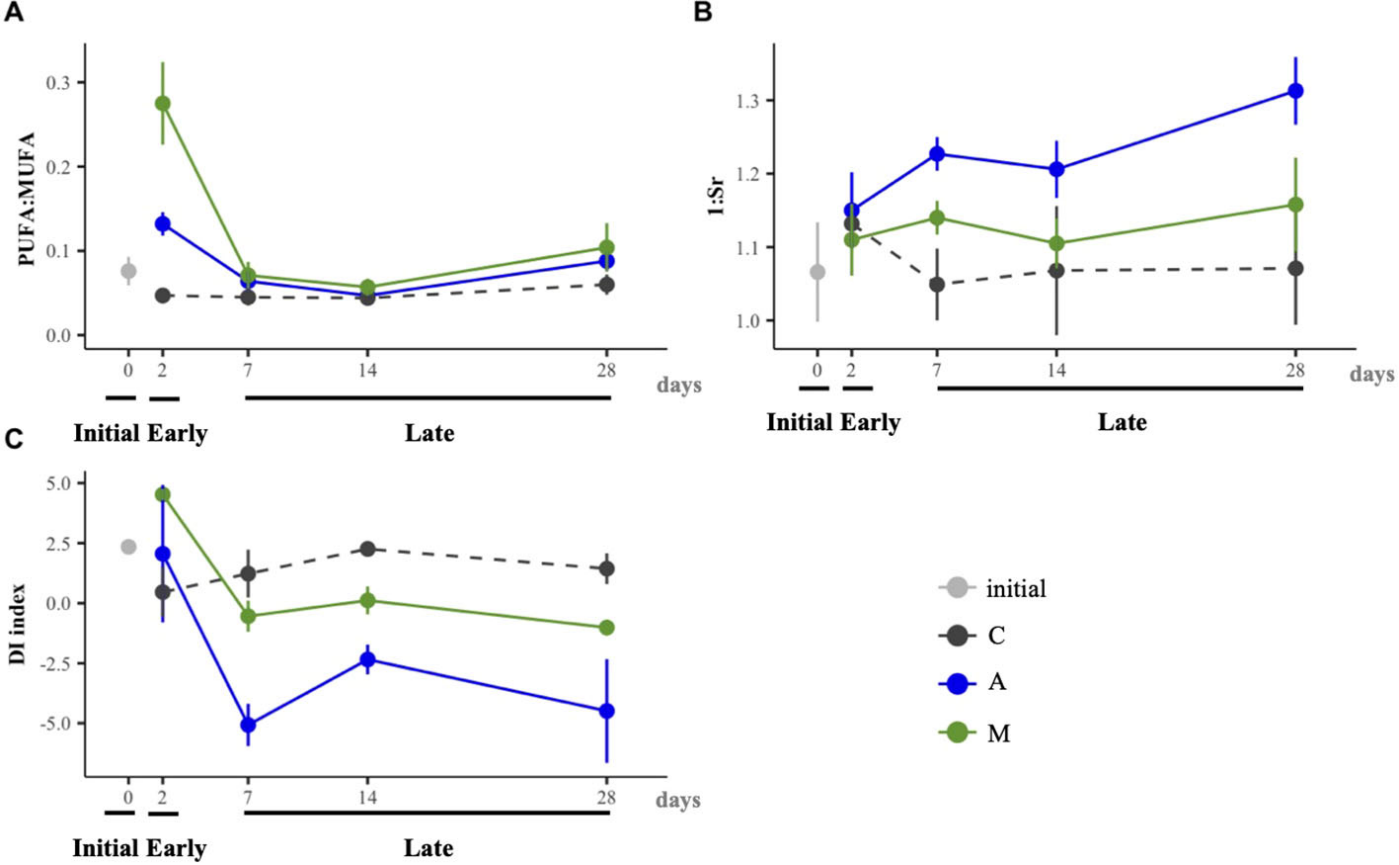
Temporal dynamic evolution of OM properties; **(A)** PUFA: MUFA ratio; **(B)** inverse of spectral slope ratio Sr (1/Sr); and **(C)** degradation index (DI) for the initial condition (gray), control condition (black dotted line), *Aphanizomenon*-derived OM (blue solid line), and *Microcystis*-derived OM (green solid line). Data are presented as mean ± standard deviation (n = 4).

Although little difference in DOC dynamics was detectable at the time steps used in this study, differences in OM chemical composition (**Figures 4**, **5**) and metabolic activities (**Supplementary Figure 7**) were observed and persisted after 28 days.

Considering all of the parameters used to characterize inorganic and organic matter (**Figure 4**), the early responses in the control and the cyanobacterial-derived OM conditions were discriminated along Axis 2 (23.5% of total inertia; **Figure 4A**). This separation was mainly due to the major contribution (>10%; **Supplementary Figure 8**) of the PUFA: MUFA ratio, BFA concentrations, OM polarity, and NH_4_^+^ concentration, all of which exhibited higher values in the cyanobacterial-derived OM conditions (**Figure 4B**). In contrast, lower SUVA_254_ values were measured in the latter conditions (**Figure 4B**). Moreover, significant differences in lability were detected (**Figures 5A, B**; p-value < 0.05 for PUFA: MUFA and DI, **Supplementary Table 7C**), with higher values of the PUFA: MUFA ratio and DI index for *Microcystis*-derived OM compared to *Aphanizomenon-*derived OM (**Figures 5A, B**; p < 0.001, **Supplementary Table 7C**).

Discrimination of the late response cyanobacterial-derived OM conditions along Axis 1 (32.1% of total inertia; **Figure 4A**) was mainly explained by higher values of NO_3_^-^ + NO_2_^-^ and PO_4_^2-^ concentrations (**Figure 4B**), the amino acids β-alanine and glycine concentrations, DOM apparent molecular weight (1/*Sr*), carbon-normalized fluorescence (Fluo/DOC), and DOC and DON concentrations. In contrast, the PUFA: MUFA ratio sharply decreased after two days and thereafter remained low and stable (from 0.04 ± 0.008 to 0.08 ± 0.011), similar to the control conditions (**Figure 5**; p > 0.05, **Supplementary Table 7C**). The DI index showed lower (p-value < 0.05, **Supplementary Table 7C**) and negative values in the cyanobacterial-derived OM conditions compared to the control samples (**Figures 4A, B**).

At the same time, the Average Well Color Development (AWCD), an overall proxy of the OM mineralization capacities at the community level (**Supplementary Figure 7**), showed higher values for the cyanobacterial-derived OM conditions for both species during the late response. AWCD values tended to return to the level of the control conditions after 28 days, but with no statistical difference (p-value > 0.05). Both the functional richness and the nitrogen use index (NUSE) tended to increase with the cyanobacterial-derived OM supply (**Supplementary Figure 7**). Only marginal changes were observed in carbon-substrate utilization profiles (**Supplementary Figure 8**), including higher values of L-arginine, L-asparagine, and L-serine (amino acids), phenylethylamine (an amine), and D-galacturonic acid and D-malic acid (carboxylic acids; **Supplementary Figure 9**).

During the late response, differential responses according to the cyanobacterial species were also detected for specific key parameters describing OM quality (**Figure 5**). The apparent molecular weight of *Aphanizomenon*-derived OM increased significantly (p-value < 0.001, **Supplementary Table 7C**), while that of *Microcystis*-derived OM remained relatively stable over time (mean value of 1.13 ± 0.02) and did not significantly differ from the control from d7 onwards (p-value > 0,05, **Supplementary Table 7C**). Moreover, lower negative DI values were observed for *Aphanizomenon*-derived OM compared to *Microcystis* during the late response (p-value < 0.01, **Supplementary Table 7C**).

To illustrate the temporal synergy between microbial communities and environmental variables, a redundancy analysis (RDA) was performed using the most explanatory variables (**Supplementary Figure 10**). The effect of cyanobacterial-derived OM was highlighted along Axis 2 (8.2% of total inertia), significantly constrained by two major characteristics: OM biodegradability (OM polarity and SUVA_254_) and dissolved nutrients (DOC, DOP, PO_4_^2-^, TDN, and TDP). The temporal responses were highlighted along Axis 1 (12.1% of total inertia), where the early-response communities were segregated from the late-response communities (according to the cyanobacteria and the lifestyle strategy), and were significantly constrained by two major characteristics indicative of OM recalcitrance during the late response: higher Fluo/DOC ratios and OM aromaticity. Moreover, the NH_4_^+^ concentrations clearly differentiated the *Aphanizomenon*- and *Microcystis*-derived OM during the early response.

## 4 Discussion

Cyanobacteria can form dense and extensive blooms in aquatic environments, releasing large amounts of organic matter (OM) and sometimes producing toxins, thereby disrupting ecosystem functioning and altering water quality (Huisman et al., 2018; Chorus et al., 2021). Although many studies have investigated such events and the interactions between OM and microbial communities (*e.g.*, Bittar et al., 2015; Ávila et al., 2019; Sandor et al., 2026), relatively few have focused on the senescent phase (Shi et al., 2017; Zhu et al., 2026). Moreover, as the senescence bloom phase corresponds to the maximal period of biogenic OM release into the environment, it provides an optimal context to investigate the interactions between biogenic OM from two distinct cyanobacterial genera and the *in vivo* heterotrophic prokaryotic community that degrades it. We thus decided to stimulate the senescent phase of two cyanobacterial blooms through the inactivation of two cyanobacterial cultures. Microwave radiation was used for inactivation in order to prevent further algal growth from our cyanobacterial cultures (Keller et al., 1988), without major changes to the molecular structure (Neas and Collins, 1988). It is known that irradiation can modify OM composition. It can cause a decrease in the higher molecular range and an increase in smaller molecules, along with an increase in the assimilable amount of organic matter (Frimmel, 1998). The organic radicals in humic and fulvic acids are reported to be sensitive to physical (UV- and VIS-radiation, X-ray, or heat) influences (Paul et al., 2006). However, compared to short wavelengths such as UV-radiation, microwaves are non-ionizing electromagnetic radiation that can increase the temperature of any absorbing medium through dipole rotation and ionic conduction, without directly altering molecular structure via photochemical bond breaking.

The synergy between the microbial compartment and OM drives a significant fraction of the energy flow in aquatic systems and biogeochemical processes (Grossart et al., 2006). Our study demonstrated that OM underwent changes that persisted for 28 days, in both OM properties and bioavailability, with responses varying according to the cyanobacterial source. These distinct OM pools did not affect the bulk microbial parameters; however, the taxonomic composition of both free-living (FL) and particle-attached (PA) fractions differed markedly. It is now well documented that PA and FL microbial communities are different in terms of taxonomic and functional composition (Ortega-Retuerta et al., 2013; Parveen et al., 2013; Liu et al., 2019; Hu et al., 2020; Louati et al., 2023; Schweitzer-Natan et al., 2023). Living in different spatial niches may provide PA microorganisms with enhanced access to resources and higher protection from grazing (Liu et al., 2019). The PA fraction could be seen as “hotspots” for mineralization with copiotroph-like bacteria, while the FL fraction could be fueled by the mineralized OM with oligotroph-like bacteria (Ortega-Retuerta et al., 2013; Koch et al., 2014). Furthermore, the fact that the bulk microbial parameters were not affected could also imply functional redundancy among the bacteria within the PA and FL fractions, allowing similar bulk functions and metabolic profiles (Louati et al., 2015; Pascault et al., 2021).

By integrating OM optical properties and biomarkers (fatty acids and amino acids), we observed that the bulk OM composition was significantly different when cyanobacterial-derived OM was added, highlighting the strong influence of the biogenic source.

After 2 days of cyanobacterial-derived OM supply, the higher PUFA: MUFA ratios suggested an early and rapid release of fresh and labile OM. At this stage, low aromaticity was detected, as evidenced by low carbon-specific UV absorbance at 254 nm (SUVA_254_ values; Helms et al., 2008; Rochelle-Newall et al., 2018) and high polarity (**Figure 4**, **Supplementary Figure 10**), reflecting a high content of hydrophilic compounds and thus a high degree of biodegradability (Grasset et al., 2023; Slentz et al., 2025). Interestingly, several OM properties varied depending on the cyanobacteria supplied. The OM was more labile when derived from senescent *Microcystis* compared to *Aphanizomenon*, as suggested by higher PUFA: MUFA ratios and DI values at d2, indicating a higher susceptibility of *Microcystis*-derived OM to early microbial degradation (Dauwe and Middelburg, 1998; Dauwe et al., 1999). The chemical profile of *Microcystis*-derived material obtained by UPLC-HRMS analyses performed by Patriarca et al. (2021) also highlighted its high lability, mainly due to a high proportion of aliphatic material with a large polarity range.

Concurrently, this release of fresh and labile OM was quickly colonized and mineralized by bacteria, as evidenced by high BFA concentrations (**Figure 4**). Indeed, the increase of these bacteria-specific FA is related to a stimulation of bacteria growth, as was also observed by Dai et al. (2009) following diatom-derived OM addition. The rapid bacterial mineralization was further supported by the high values of exo-proteolytic activity (EPA) at d2 together with increased DIN concentrations (**Supplementary Figure 6**). Recent studies suggest that extracellular enzyme production can be stimulated when OM is largely composed of fresh and labile compounds (Baltar et al., 2017; David et al., 2021), consistent with previous reports of highly efficient bacterial utilization of phytoplanktonic lysates (Dai et al., 2009; Attermeyer et al., 2014; Landa et al., 2014; Shen and Benner, 2020).

Different OM sources supported different microbial communities (**Supplementary Figure 10**), and these differences could be attributed to the ability of bacteria to utilize different substrates (Sandor et al., 2026). During the early-response phase, the increase in Alphaproteobacteria taxa may have been triggered by the supply of labile material, which was rapidly consumed (Berg et al., 2018). Interestingly, the emerging Alphaproteobacteria taxa identified here differ from those selected during the bloom event in the phycosphere (growth phase). Previous studies have highlighted the emergence of Nitrosomonadaceae and Comamonadaceae in the PA fraction (Shao et al., 2014; Louati et al., 2015, 2023; Nelson et al., 2021; Pascault et al., 2021; Fortin et al., 2022).

During the late-response phase, the proportion of recalcitrant, complex, and stable organic compounds increased when cyanobacterial-derived OM was supplied (**Figures 4**, **5**, and **S10**). These shifts in OM reactivity, from labile at the early stage to recalcitrant, stable, and persistent forms at late stages, have also been observed in freshwater mesocosms following *Microcystis*-derived OM degradation and were attributed to gradual processing by heterotrophic bacteria and bacterial succession (Patriarca et al., 2021; Freeman et al., 2024; Chen et al., 2025). Laboratory and bioassay experiments have shown that natural marine bacterial communities can rapidly transform labile substrates into refractory DOM that persists for extended periods, underscoring the key role of bacteria in producing chemically complex and persistent molecules from simple organic compounds (Ogawa, 2001; Lechtenfeld et al., 2015; Zhao et al., 2019; Sandor et al., 2026). In lake ecosystems, the persistence of DOM has been shown to be closely linked to its molecular characteristics and properties, with degradation processes preferentially removing oxidized and aromatic compounds, while reduced, aliphatic, and nitrogen-containing compounds tend to resist degradation, thereby persisting in aquatic systems (Kellerman et al., 2015). Why and how DOM persists is still a topic of debate across ecosystems: in soils (Zsolnay, 2003; Gmach et al., 2020), freshwater (Kellerman et al., 2018), and in oceans (Koch et al., 2014; Osterholz et al., 2015; Dittmar et al., 2021). However, our study provides evidence on the microbial carbon pump, highlighting the pivotal role of microbial-mediated processes in converting readily bioavailable, autochthonous OM into chemically recalcitrant OM pools.

Differences in properties and reactivity between the cyanobacterial-derived OM pools persisted 28 days after senescent OM supply. Indeed, the *Aphanizomenon-*derived OM exhibited particularly low negative DI values and greater apparent molecular weight than *Microcystis-*derived OM, suggesting distinct compositional and structural characteristics influencing OM biodegradability, with more degraded, recalcitrant, and complex OM (Davis et al., 2009; Peter et al., 2012; Shen and Benner, 2020) when it comes from *Aphanizomenon* as compared to *Microcystis*.

During these late responses, the distinct and long-lasting differences in the compositional and reactive characteristics of the two cyanobacterial-derived OM pools did not influence the bacterial exo-proteolytic activities or metabolic profiles. However, previous studies have shown that substrate utilization patterns (Biolog ECOPLATES^®^) can be employed to provide insights into the functional trait structure underlying bacterial OM mineralization and their relationship with OM quality (Ruiz-González et al., 2015). Similarly, only limited differences were observed in the abundance of prokaryotes, potentially reflecting top-down control by viruses and/or micro-eukaryotes (**Supplementary Figure 2**). A grazing response following a natural cyanobacterial bloom was also observed in field conditions (Sellner et al., 1993; Work and Havens, 2003; Berg et al., 2018).

The more degraded and more complex derived-OM from *Aphanizomenon* at the late-response stage suggested a larger fraction of molecules that require different and specific enzymatic machineries, thereby favoring bacteria that possess specific metabolic capabilities (Landa et al., 2014). Within the *Aphanizomenon*-derived OM conditions, we detected an increase in abundance of Sphingomonadales and Burkholderiales. These groups are known for their ability to metabolize recalcitrant and complex compounds, including humic-like and aromatic fractions (Ma et al., 2020; Fagervold et al., 2023). Finally, while a return to the initial conditions after 7 days was detected at the community level, a specific Bacilli group emerged and persisted over time only in the *Microcystis*-derived OM conditions. Members of Firmicutes were previously identified as enhanced taxa within the *Microcystis* phycosphere during bloom events (Liu et al., 2019; Pascault et al., 2021; Chen et al., 2025), while they represented a minor fraction under non-bloom conditions (Mankiewicz-Boczek and Font-Nájera, 2022; Van Le et al., 2024). Moreover, they are associated with the mineralization of *Microcystis* scum, due to their high hydrolytic enzymatic potential (Xing et al., 2011; Liu et al., 2019; Dai et al., 2022; Martínez-García et al., 2022; Trano et al., 2022), and thus may be linked to lake water quality deterioration. Although they are not known to remain abundant in aquatic ecosystems, in this study, they persisted and were still abundant after 28 days.

Altogether, these results highlight that the senescence of cyanobacterial blooms leaves both a chemical and a bacterial footprint on lake ecosystems, emphasizing the crucial role of cyanobacterial-derived OM and the connection between chemo-diversity and bacterial diversity (Tanentzap et al., 2019; Zhao et al., 2019). Each bloom could result in long-term chemical and biological disruption, characterized by the accumulation of refractory OM and different resilience trajectories depending on the cyanobacterial species. As a central component of biogeochemical cycles, the persistence of recalcitrant organic matter following cyanobacterial blooms has broad-scale implications for carbon cycling and storage in lake ecosystems. The two cyanobacterial-derived OM pools displayed distinct contrasting bioavailability profiles and reactivity patterns, with different properties that persisted over time, reflecting stable source-specific properties. Furthermore, previous studies have highlighted a potential year-to-year drift in community composition in hypereutrophic lakes (Kraemer et al., 2020; Jiao et al., 2021; Duperron et al., 2026), resulting from a long-term potential impact of cyanobacterial blooms on the dynamics of OM cycling and the structural composition of microbial communities. Herein, the bacterial footprint was identified as the emergence and the persistence of Bacilli members, a possible indicator of lake ecosystem health. Coupling isotope labelling of organic matter (Kwon et al., 2026) with the manipulation of synthetic communities (Kim et al., 2021) could provide an opportunity to better decipher the synergistic link between OM pools and the microbial compartment.

## Data Availability

The original contributions presented in the study are publicly available. This data can be found here: NCBI, accession PRJNA1456822.
