# Supplementary material for: Synergistic interactions between biogenic organic matter and microbial dynamics during simulated senescent cyanobacterial blooms in freshwater mesocosms
Source: Front Microbiomes. 2026 Jul 13;5:1866641. doi: 10.3389/frmbi.2026.1866641 (PMC13402508; doi:10.3389/frmbi.2026.1866641)

Supplementary material

List of supplementary tables (see Excel file "Suppl_Tables")

<u>Table S1.</u> Chemical composition of the inactivated cyanobacterial cultures

Concentrations are given as µM, in the culture medium, and after dilution in the mesocoms (Vf = 15L)

<u>Table S2.</u> Samples nomenclature and bio-informatics pre-processes

<u>Tables S3.</u> Statistical analysis of the ordination of the particle-associated (PA) and the free-Living (FL) microbial communities (see Figure 1). PERMANOVA results are presented for both the PA and FL communities together, pairwise analysis is presented separately for the FL communities (B) and PA communities.

<u>Table S4.</u> Statistical analysis (lme models) of the diversity indexes of the particle-associated (PA) and the free-Living (FL) microbial communities

<u>Tables S5.</u> Taxonomic composition of the microbial communities at the class level. Data are presented as relative abundance of the total reads (percentage), A) within the free-living fraction and B) within the particle-associated fraction. Statistical analyses on the abundances of the taxonomical classes in the FL (C and D) and PA (E and F) microbial communities; according to the derived-OM supply and the response-time (C and E); and according to the cyanobacterial species for the derived-OM supply (D and F).

<u>Tables S6.</u> Summary of the 45 ASVs (contributing to up to 70% of the cumulative explained dissimilarity (p-value <0.01) between the cyanobacterial-derived OM conditions and the time-responses within the PA fractions, SIMPER analysis). A differential analysis (DESeq) was performed in order to describe three spatial patterns (-2 < LogFold change (LFC) > 2, with an adjusted p-value < 0.001).

<u>Tables S7.</u> Statistical analyses (lme models) for the inorganic (A) and organic nutrients (B), OM concentration and quality (C).

List of supplementary figures

Figure S1: Similarity profile analysis (SIMPROF) of the microbial communities from the free-living (FL) and particle-associated (PA) fractions, according to the time-responses (Initial, d2, d7, d14 and d28 days) and the *Aphanizomenon* (A) and *Microcystis* (M) derived-OM supply. The dendrogram highlights significant colored clusters (p-value<0.05), based on a Bray-Curtis distance matrix.

Figures S2. Alpha-diversity indexes of the bacterial communities from (A) the free-living (FL) and (B) the particle-associated (PA) fractions for the control conditions (grey), *Aphanizomenon*- (blue), *Microcystis*- (green) derived OM conditions, and according to the Early- and Late-responses. Data are presented as boxplot (n=4).

Figure S3. Temporal dynamics of the microbial abundances for the initial conditions (grey), control conditions (black-dashed line), *Aphanizomenon*- (blue line), *Microcystis*- (green line) derived organic matter, and according to the Early- and Late-responses. A) Prokaryotes, B) Micro-eukaryotes, C) Virus-Like Particles (VLP), D) ratio VLP:Prokaryotes, and E) ratio VLP:Micro-eukaryotes. Data are presented as mean values +/- standard deviation (n=4).

Figure S4. Taxonomic composition of the bacterial communities from the particle-associated fractions of *Aphanizomenon*- (A), *Microcystis*- (M) cultures, and according to the time-response (Initial, Early- and Late-responses) and the cyanobacterial-derived OM conditions; *Aphanizomenon*- (A), *Microcystis*- (M) and Control (C). Data are presented as relative abundances (%) of the dominant ASVs (> 0.1% of the whole dataset) at the Class level.

Figure S5. Temporal dynamic of the microbial communities. NMDS analysis was performed on both bacterial communities from the cyanobacterial derived-OM of *Aphanizomenon*- (blue) and *Microcystis*- (green) (PA_Culture), the free-living (FL, dot) and particle-associated (PA, triangle) fractions for the control conditions (grey), *Aphanizomenon*- (blue), *Microcystis*- (green) derived OM conditions, and according to the Initial (solid line), Early- (dashed line) and Late- (long-dashed line) responses.

Figures S6. Temporal dynamic of the dissolved organic and inorganic nutrients A) Dissolved Organic Carbon (DOC), B) Dissolved Organic Nitrogen (DON), C) Dissolved Organic Phosphorous, D) Nitrate and nitrite (NO_3^--NO_2^-) concentrations, (E) Ammonium (NH_4^+) concentration, and (F) Phosphate (PO_4^{2-}) concentration, for the initial conditions (grey), control conditions (black dotted-line), *Aphanizomenon*- (blue solid line), *Microcystis*- (green solid line) derived OM conditions. Data are presented as mean values ± standard deviation (n = 4).

Figures S7. Temporal dynamic of potential enzymatic activities, A) Exo-proteolytic activities, and B) Average well color development (AWCD), C) the functional richness, and D) the

Nitrogen Yse index (NUSE) for the initial conditions (grey), control conditions (black dotted-line), *Aphanizomenon*- (blue solid line), *Microcystis*- (green solid line) derived OM conditions. Data are presented as mean values ± standard deviation (n = 4)

Figure S8. Contribution of quantitative variables to the first dimension (A) and the second dimension (B) of the MFA analysis. The red dashed line corresponds to the average expected contribution of the 17 variables (1/17*100 = 5,88 %).

Figure S9. Temporal dynamic of each metabolic substrate within amino-acids, amines, carboxylic acids, carbohydrates, phenolic compounds and polymers families. Data are presented as mean values of normalized AWCD (± standard deviation, n = 4); for the initial conditions (grey), control conditions (black dotted-line), *Aphanizomenon*- (blue solid line), *Microcystis*- (green solid line) derived OM conditions.

Figure S10: Redundancy analysis of the microbial communities, with the environmental variables (significantly explaining variables, adjusted p-value<), and the Top-20 ASVs (significantly explaining ASVs), from the free-living (FL, dot) and particle-associated (PA, triangle) fractions for the control conditions (grey), *Aphanizomenon*- (blue), *Microcystis*- (green) derived OM conditions, and according to the Initial (plain shapes), Early- (open shapes) and Late- (plain shapes) responses; (DOC) Dissolved Organic Carbon, (DOP) Dissolved Organic Phosphorus, (TDN) Total dissolved Nitrogen, (TDP) Total dissolved Phosphorus, (NH_4^+) ammonium concentration, (PO_4^{2-}) phosphate concentration, OM aromaticity, $SUVA_{254}$ index, and FLUO:DOC ratio.

Figure S1: Similarity profile analysis (SIMPROF) of the microbial communities from the free-living (FL) and particle-associated (PA) fractions, according to the time-responses (Initial, d2, d7, d14 and d28 days) and the *Aphanizomenon* (A) and *Microcystis* (M) derived-OM supply. The dendrogram highlights significant colored clusters (p-value<0.05), based on a Bray-Curtis distance matrix.

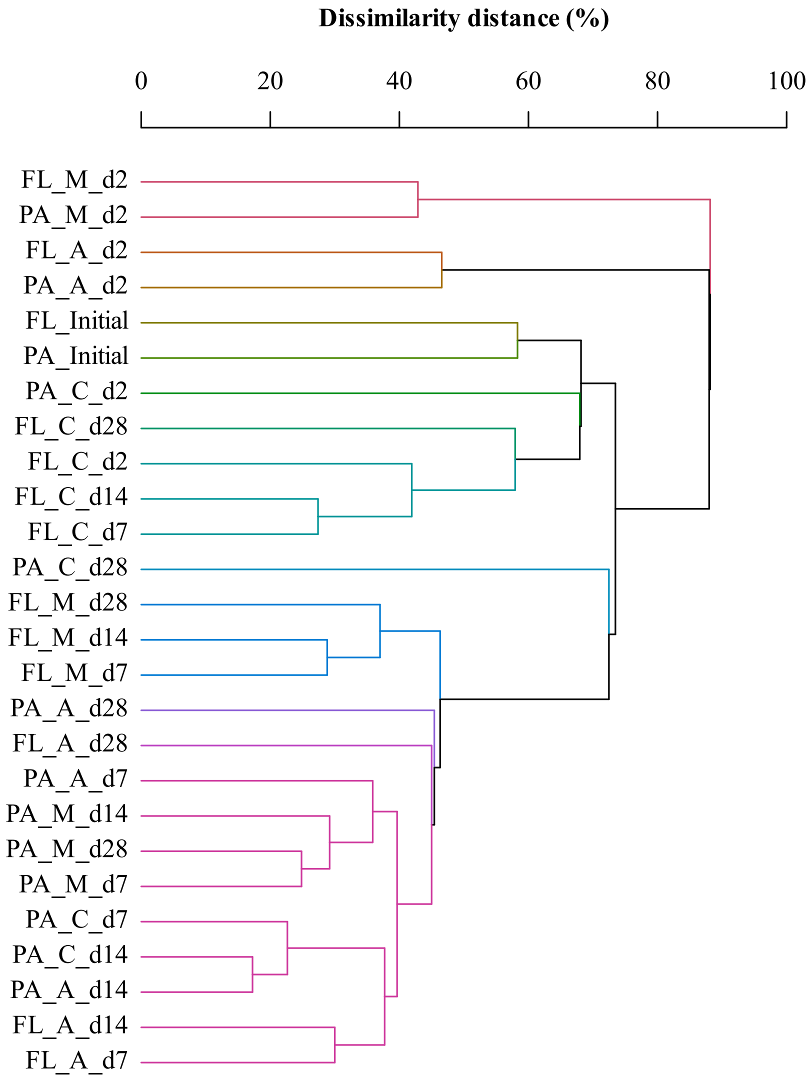

Figure S2. Alpha-diversity indexes of the bacterial communities from (A) the free-living (FL) and (B) the particle-associated (PA) fractions for the control conditions (grey), *Aphanizomenon*-(blue), *Microcystis*- (green) derived OM conditions, and according to the Early- and Late-responses. Data are presented as boxplot (n=4).

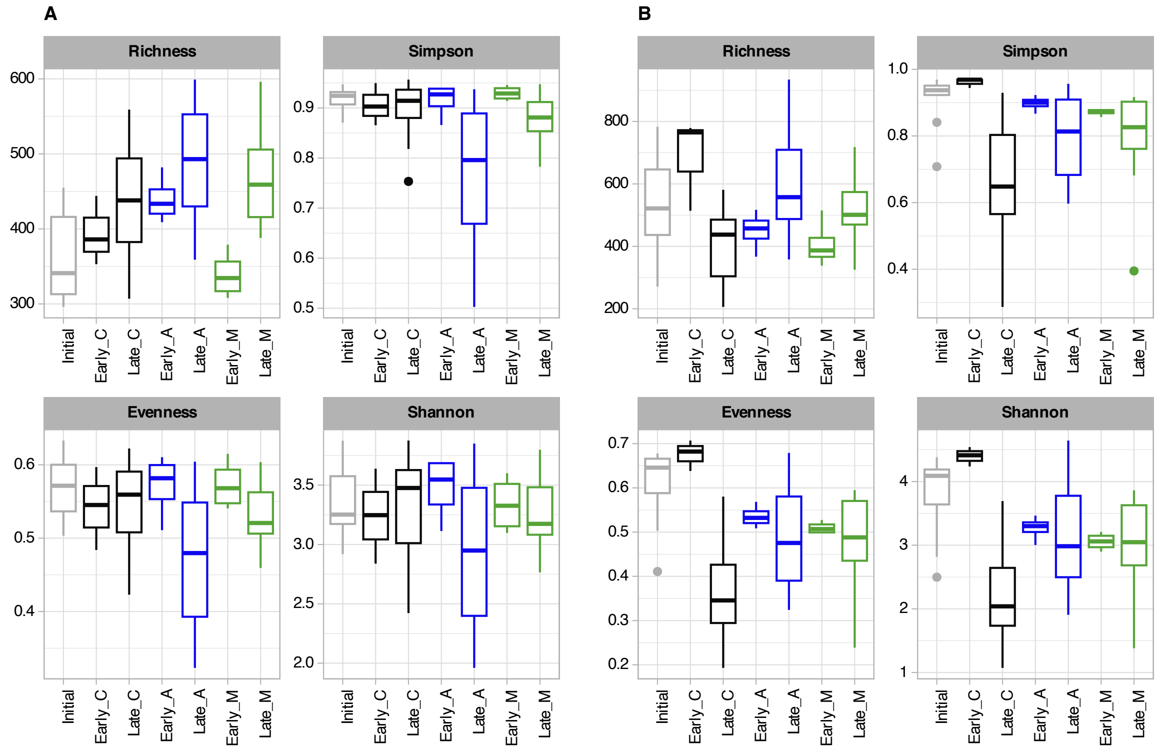

Figures S3.

Temporal dynamics of the microbial abundances for the initial conditions (grey), control conditions (black-dashed line), *Aphanizomenon*- (blue line), *Microcystis*- (green line) derived organic matter, and according to the Early- and Late-responses. A) Prokaryotes, B) Micro-eukaryotes, C) Virus-Like Particles (VLP), D) ratio VLP:Prokaryotes, and E) ratio VLP:Micro-eukaryotes. Data are presented as mean values +/- standard deviation (n=4).

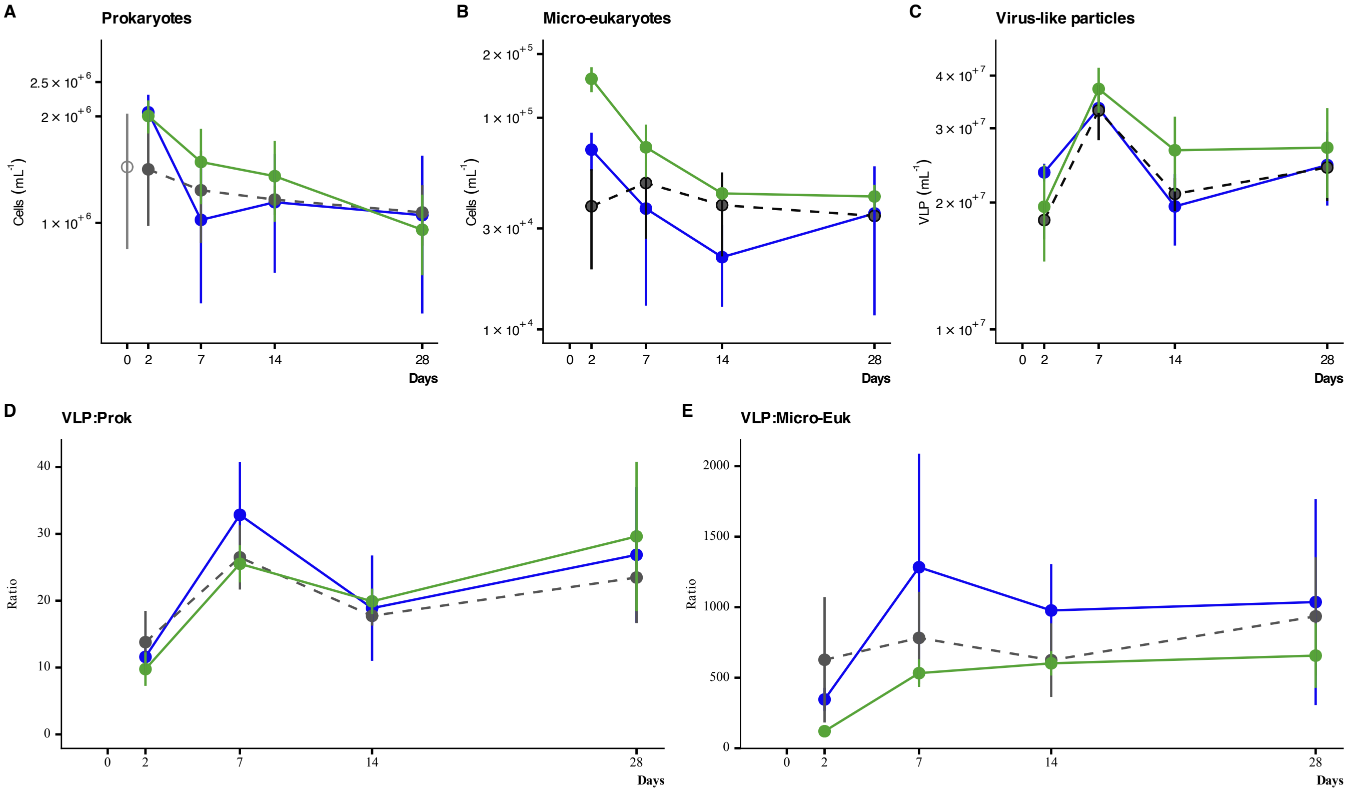

Figure S4. Taxonomic composition of the bacterial communities from the particle-associated fractions of *Aphanizomenon*- (A), *Microcystis*- (M) cultures, and according to the time-response (Initial, Early- and Late-responses) and the cyanobacterial-derived OM conditions; *Aphanizomenon*- (A), *Microcystis*- (M) and Control (C). Data are presented as relative abundances (%) of the dominant ASVs (> 0.1% of the whole dataset) at the Class level.

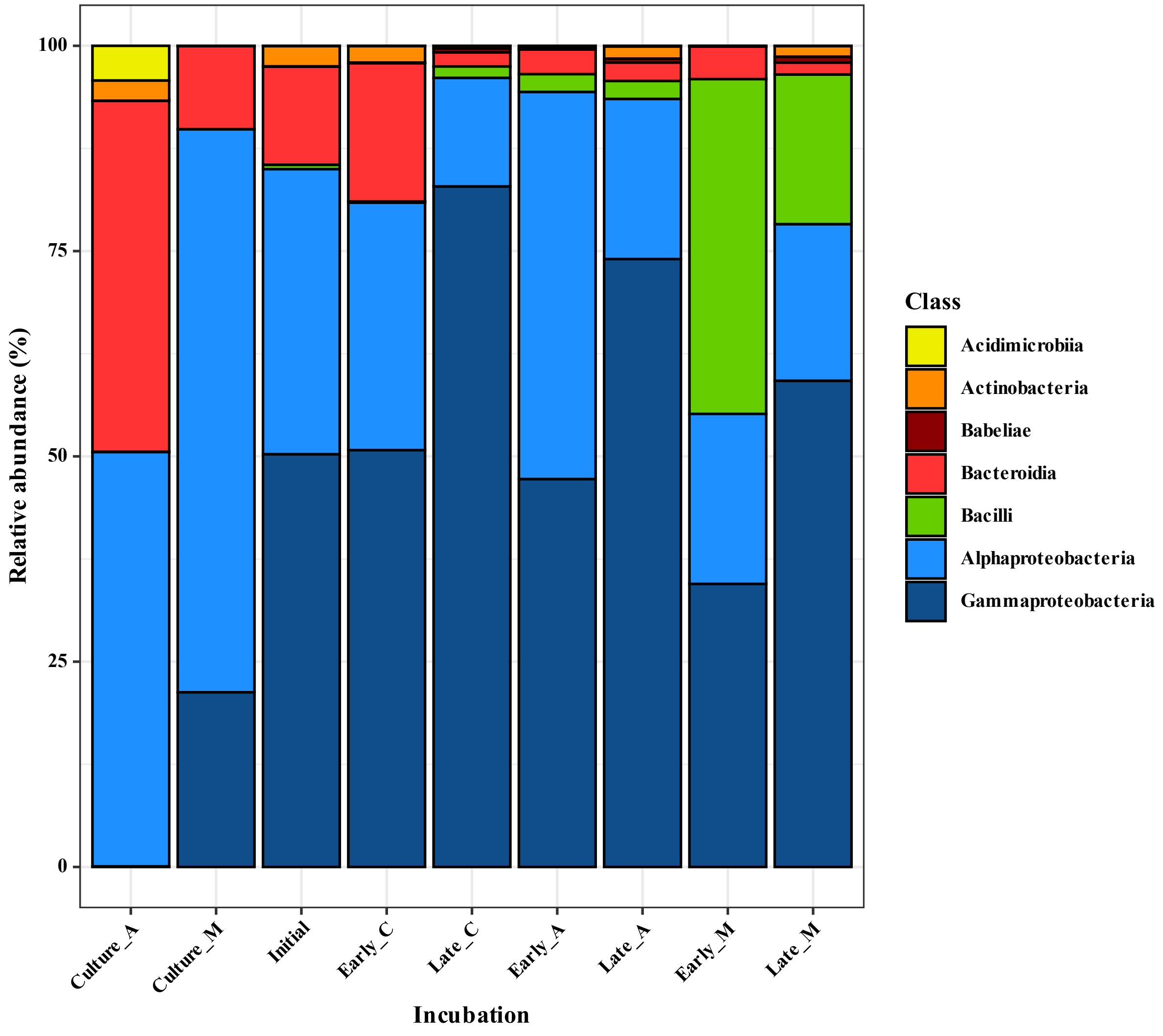

Figure S5. Temporal dynamic of the microbial communities. NMDS analysis was performed on both bacterial communities from the cyanobacterial derived-OM of *Aphanizomenon*- (blue) and *Microcystis*- (green) (PA_Culture), the free-living (FL, dot) and particle-associated (PA, triangle) fractions for the control conditions (grey), *Aphanizomenon*- (blue), *Microcystis*- (green) derived OM conditions, and according to the Initial (solid line), Early- (dashed line) and Late- (long-dashed line) responses. In the spider-plot, the forms correspond to the position of the centroid of the conditions, and the end of the lines correspond to the position of each sample.

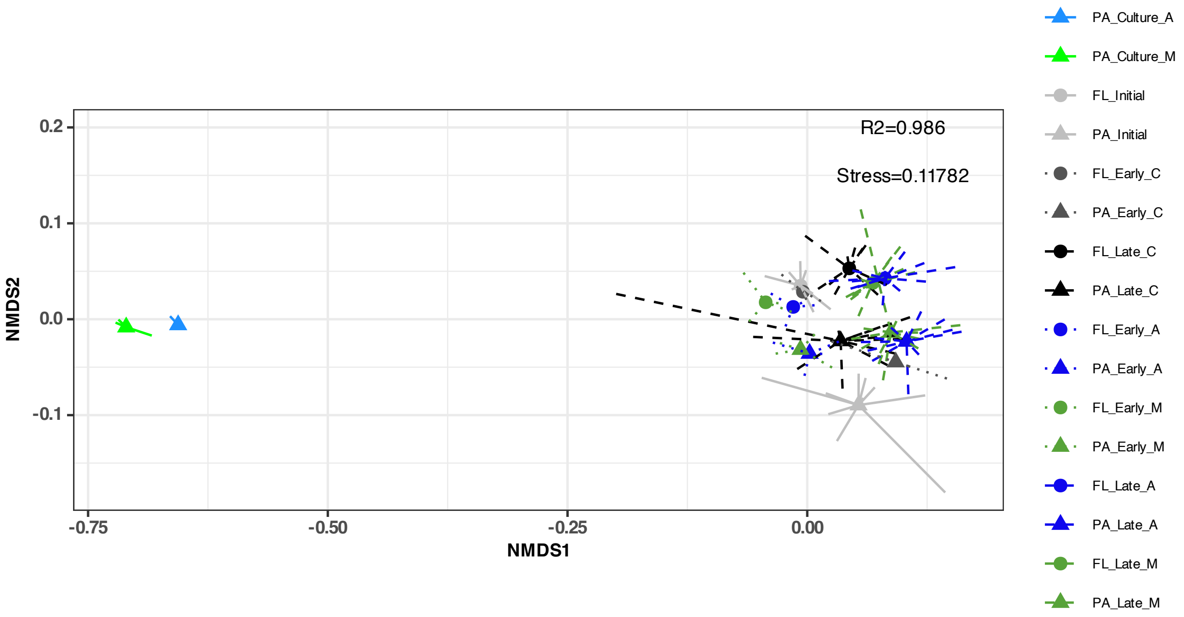

<u>Figure S6.</u> Temporal dynamic of the dissolved organic and inorganic nutrients A) Dissolved organic Carbon (DOC), B) Dissolved organic Nitrogen (DON), C) Dissolved organic Phosphorous, D) Nitrate and nitrite (NO_3^--NO_2^-) concentrations, (E) Ammonium (NH_4^+) concentration, and (F) Phosphate (PO_4^{2-}) concentration, for the initial conditions (grey), control conditions (black dotted-line), *Aphanizomenon*- (blue solid line), *Microcystis*- (green solid line) derived OM conditions. Data are presented as mean values ± standard deviation (n = 4).

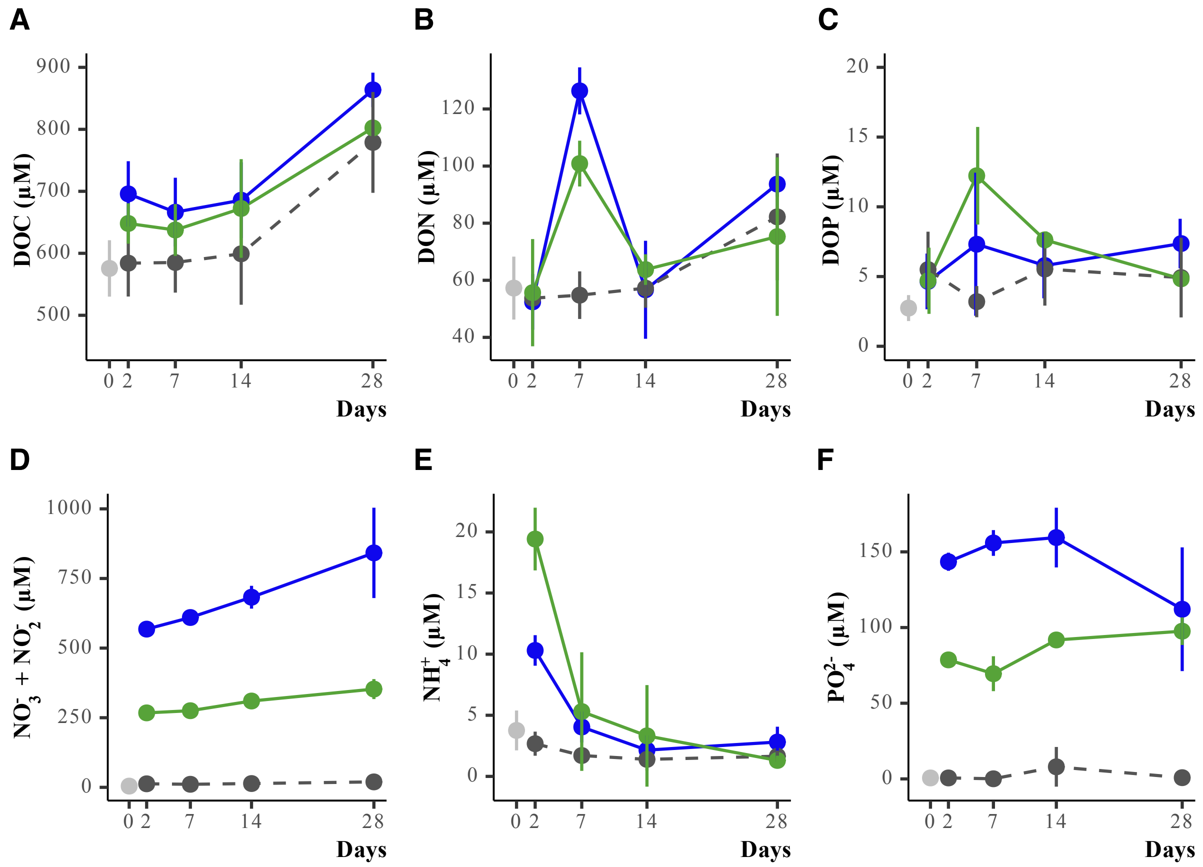

Figures S7. Temporal dynamic of potential enzymatic activities, A) Exo-proteolytic activities, and B) Average well color development (AWCD), C) the functional richness, and D) the nitrogen use index (NUSE) for the initial conditions (grey), control conditions (black dotted-line), *Aphanizomenon*- (blue solid line), *Microcystis*- (green solid line) derived OM conditions. Data are presented as mean values ± standard deviation (n = 4)

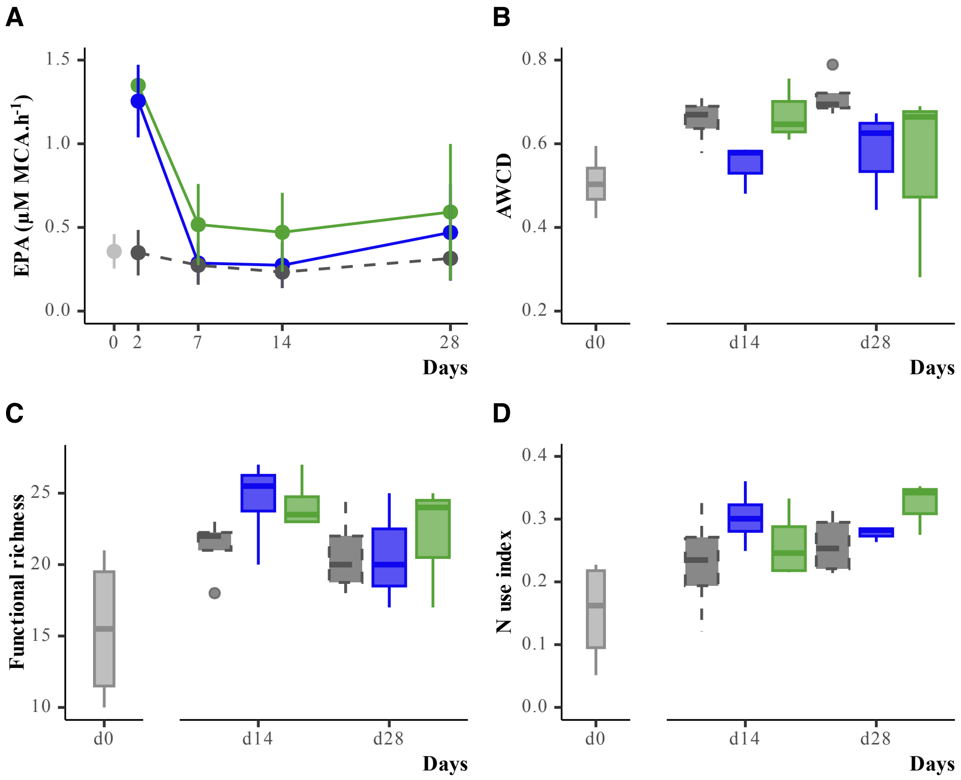

<u>Figure S8.</u> Contribution of quantitative variables to the first dimension (A) and the second dimension (B) of the MFA analysis. The red dashed line corresponds to the average expected contribution of the 17 variables (1/17*100 = 5,88 %).

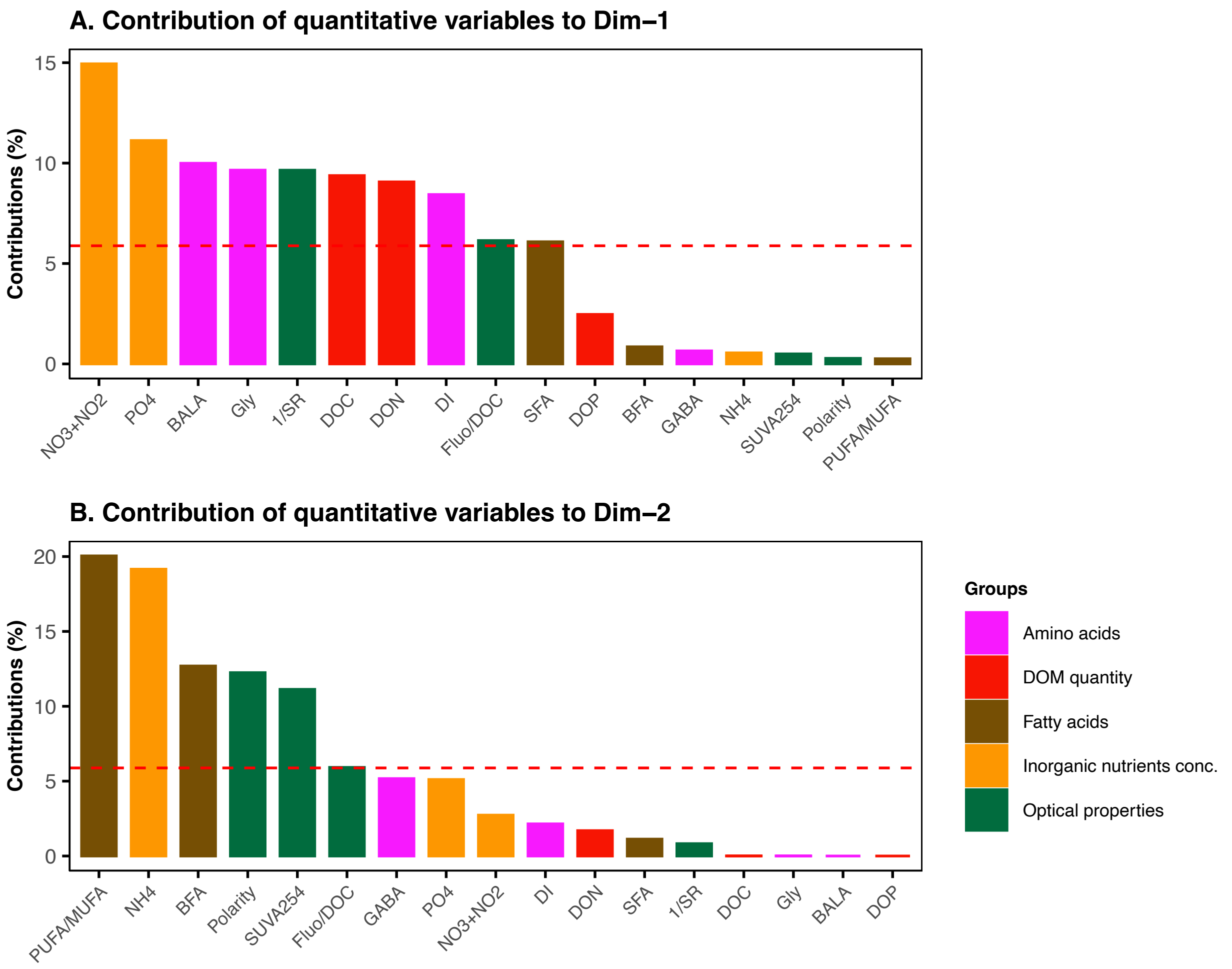

<u>Figure S9.</u> Temporal dynamic of each metabolic substrate within amino-acids, amines, carboxylic acids, carbohydrates, phenolic compounds and polymers families. Data are presented as mean values of normalized AWCD (± standard deviation, n = 4); for the initial conditions (grey), control conditions (black dotted-line), *Aphanizomenon*- (blue solid line), *Microcystis*- (green solid line) derived OM conditions.

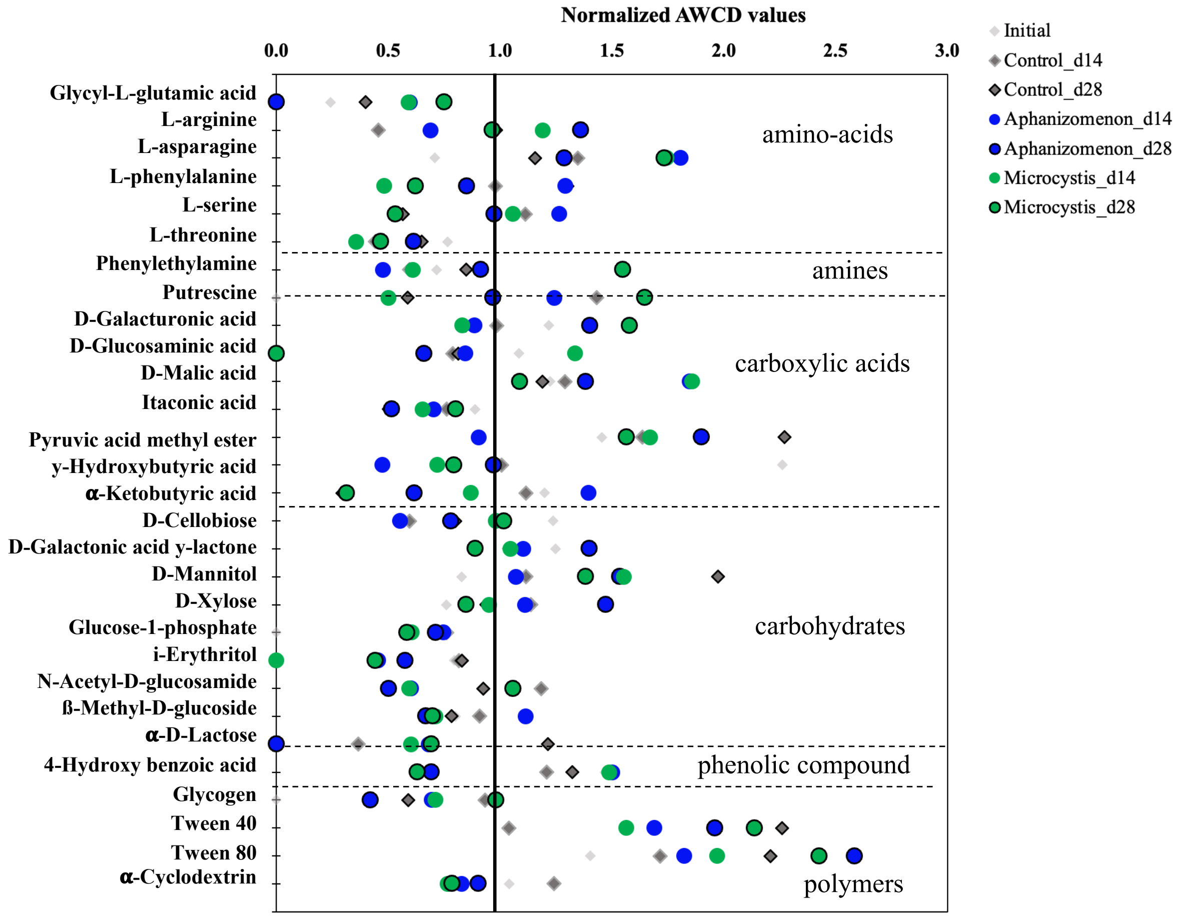

<u>Figure S10:</u> Redundancy analysis of the microbial communities, with the environmental variables (significantly explaining variables, adjusted p-value<), and the Top-20 ASVs (significantly explaining ASVs), from the free-living (FL, dot) and particle-associated (PA, triangle) fractions for the control conditions (grey), *Aphanizomenon*- (blue), *Microcystis*- (green) derived OM conditions, and according to the Initial (plain shapes), Early- (open shapes) and Late- (plain shapes) responses; (DOC) Dissolved Organic Carbon, (DOP) Dissolved Organic Phosphorus, (TDN) Total dissolved Nitrogen, (TDP) Total dissolved Phosphorus, (NH_4^+) ammonium concentration, (PO_4^{2-}) phosphate concentration, OM aromaticity, $SUVA_{254}$ index, and FLUO:DOC ratio.

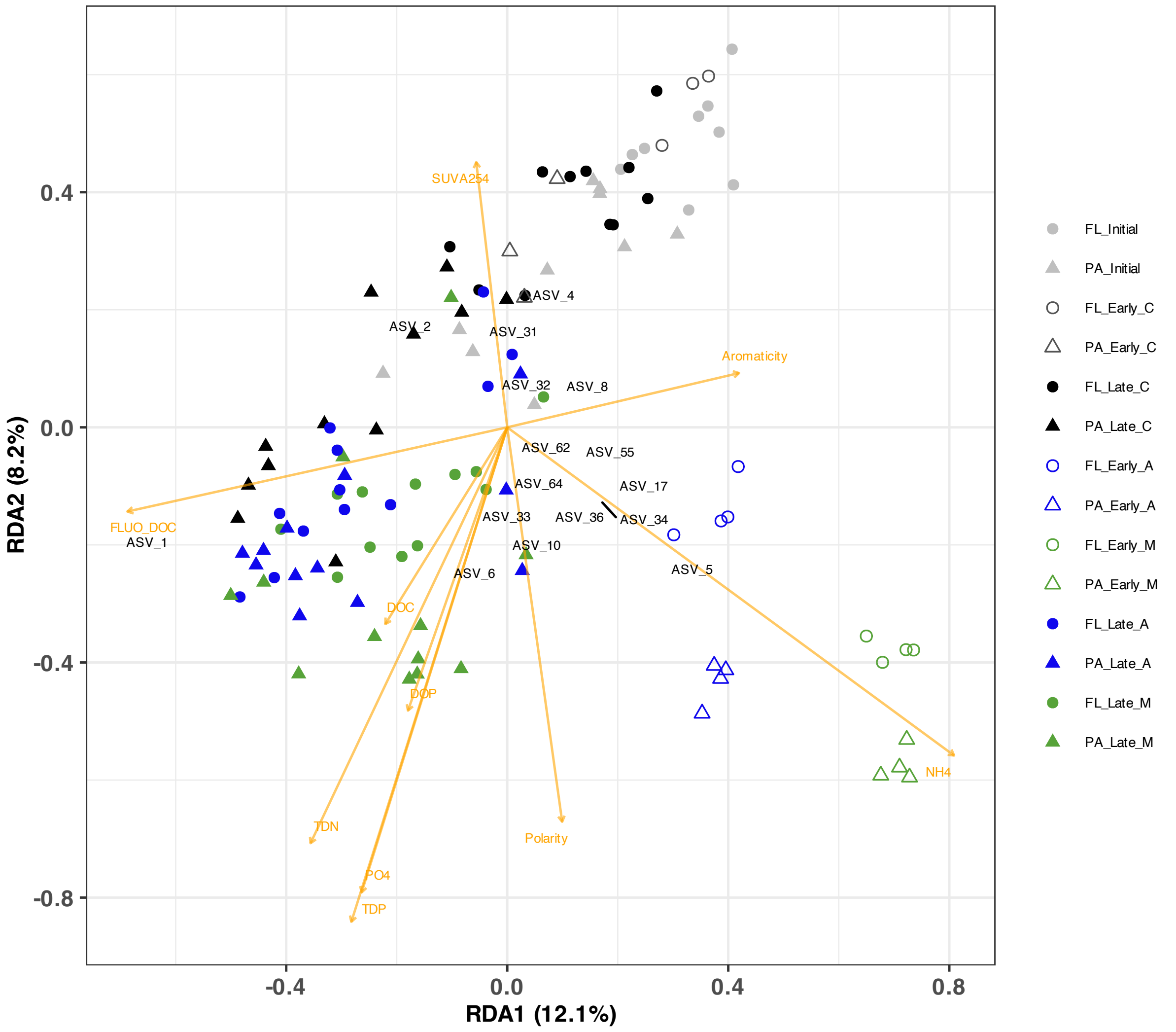

Supplement: Supplementary file 1 [file Supplementaryfile1.pdf]
